# Functional ecological convergence between the thylacine and small prey-focused canids

**DOI:** 10.1186/s12862-021-01788-8

**Published:** 2021-04-21

**Authors:** Douglass S. Rovinsky, Alistair R. Evans, Justin W. Adams

**Affiliations:** 1 Department of Anatomy and Developmental Biology, Biomedicine Discovery Institute, Monash University, Clayton, VIC Australia; 2 School of Biological Sciences, Monash University, Clayton, VIC Australia; 3 Geosciences, Museums Victoria, Melbourne, VIC Australia

**Keywords:** Thylacine, Tasmanian tiger, Convergent evolution, Prey size, Geometric morphometrics, Feeding ecology, Functional ecology

## Abstract

**Background:**

Morphological convergence is a fundamental aspect of evolution, allowing for inference of the biology and ecology of extinct species by comparison with the form and function of living species as analogues. The thylacine (*Thylacinus cynocephalus*), the iconic recently extinct marsupial, is considered a classic example of convergent evolution with the distantly related placental wolf or dog, though almost nothing is actually known regarding its ecology. This lack of data leads to questions regarding the degree of convergence with, and the similarity of, the functional ecology of the thylacine and the wolf/dog. Here, we examined the cranium of the thylacine using 3D geometric morphometrics and two quantitative tests of convergence to more precisely determine convergent analogues, within a phylogenetically informed dataset of 56 comparative species across 12 families of marsupial and placental faunivorous mammals. Using this dataset, we investigated patterns of correlation between cranial shape and diet, phylogeny, and relative prey size across these terrestrial faunivores.

**Results:**

We find a correlation between cranial, facial, and neurocranial shape and the ratio of prey-to-predator body mass, though neurocranial shape may not correlate with prey size within marsupials. The thylacine was found to group with predators that routinely take prey smaller than 45% of their own body mass, not with predators that take subequal-sized or larger prey. Both convergence tests find significant levels of convergence between the thylacine and the African jackals and South American ‘foxes’, with lesser support for the coyote and red fox. We find little support for convergence between the thylacine and the wolf or dog.

**Conclusions:**

Our study finds little support for a wolf/dog-like functional ecology in the thylacine, with it instead being most similar to mid-sized canids such as African jackals and South American ‘foxes’ that mainly take prey less than half their size. This work suggests that concepts of convergence should extend beyond superficial similarity, and broader comparisons can lead to false interpretations of functional ecology. The thylacine was a predator of small to mid-sized prey, not a big-game specialist like the placental wolf.

**Supplementary Information:**

The online version contains supplementary material available at 10.1186/s12862-021-01788-8.

## Background

Convergent evolution—the independent acquisition of similar phenotypes by separate lineages—is a fundamental and often striking aspect of biology. This repeated appearance of similar phenotypes is often assumed to result from similar adaptive processes [1–3], leading to the assumption that the similar morphologies reflect comparable functionalities. An effect of this pattern is the ability to infer function from morphology in extinct species, and by extension, selective pressures the extinct species were subject to [4, 5]. Inferring the function and ecology of an extinct species, however, can be less straightforward than a one-to-one mapping of analogous form to function. Detailed investigation reveals that functional convergence does not always produce morphological similarity [‘many-to-one’ mapping; 6,7], and conversely that morphological similarity does not always produce functional convergence [‘one-to-many’ mapping; 8–10]. Attempting to infer the functional ecology of an extinct species by analogy with living examples, even if morphologically similar, requires a careful understanding of the living analogues, as morphologically similar and even closely related animals can be highly ecologically disparate.

**Fig. 1:**
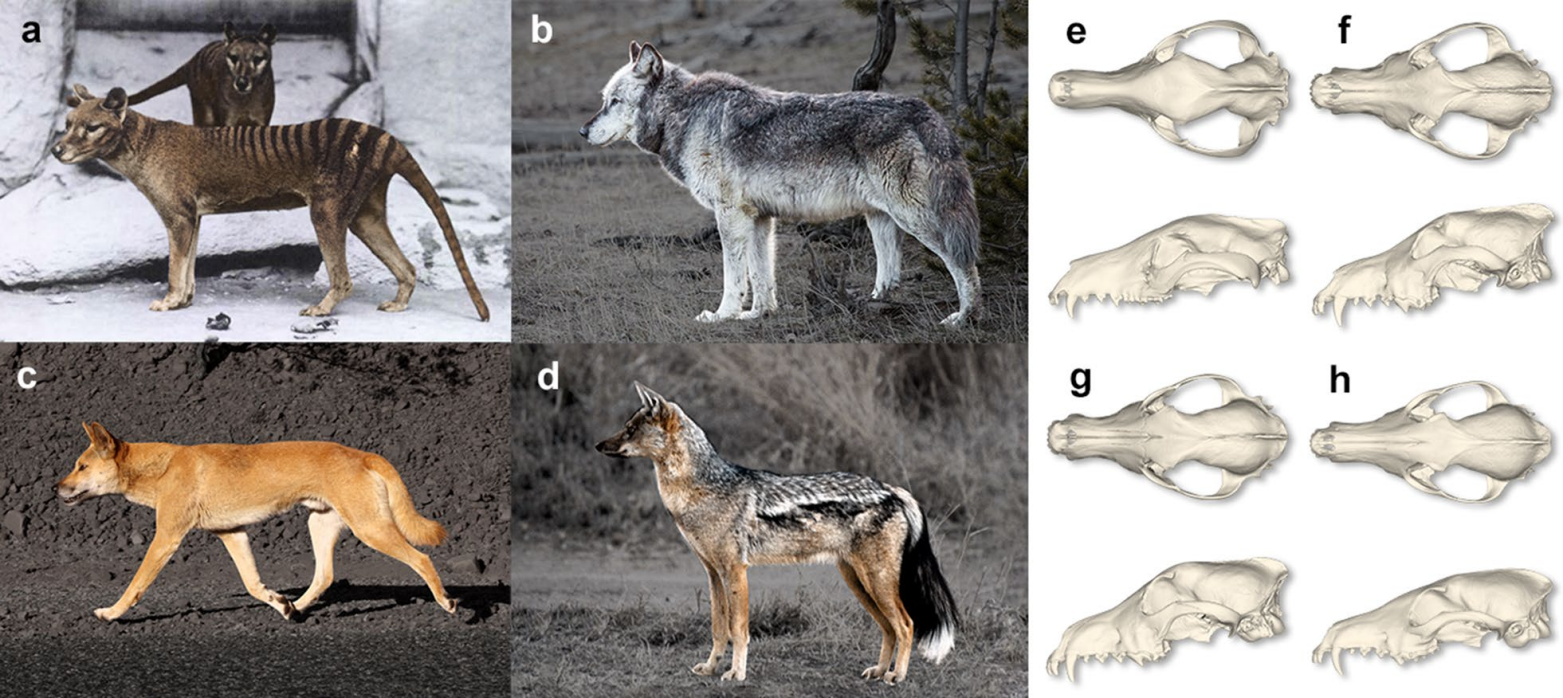
The thylacine and canid comparatives. Photographs and crania in dorsal and lateral view of **a**, **e** thylacine (*Thylacinus cynocephalus*), **b**, **f** gray wolf (*Canis lupus*), **c**, **g** dingo, and **d**, **h** side-striped jackal (*Lupulella adustus*). Images not to scale. Image credits: **a** by E.J.K. Baker, Report of the Smithsonian Institution 1904, public domain, colourised by the authors; **b** by Neil Herbert, Yellowstone National Park, public domain; **c** by Jarrod Amoore CC BY 2.0; **d** by T.A. Hermann, NBII WikiCommons, public domain. Images have been adjusted to enhance contrast between subject and background by the authors. Crania **e**–**h** are mean shape mesh warps derived from this study’s dataset

The thylacine (*Thylacinus cynocephalus*; Fig. 1a) was the largest marsupial predator to persist into modern times [11, 12]. Once widespread throughout Australia and New Guinea [13], by around 3200 years ago it was restricted to a single population on the island of Tasmania [14], where it was first encountered by European colonists in the early 19th Century [15]. By 1840, a bounty was placed on the thylacine due to fears that it was preying on livestock [16], and within 100 years the last known thylacine died in a zoo in Hobart, Tasmania, in 1936. Though the extinction of the thylacine was recent enough for a filmed record of the animal (but only in captivity), virtually no observational data exists, and as a result, we know very little regarding its functional ecology outside of anecdotal reports. This leaves us reliant on comparison with its convergent analogues to understand its functional ecology.

The external similarity between the thylacine and the gray wolf/dog complex (*Canis lupus *sensu* amplo*; Fig. 1b, c) has led to the thylacine to be considered a striking example of convergent evolution between distantly related clades [17–25]. The superficial similarity between this marsupial carnivore and the gray wolf/dog species complex is echoed in the modern day common names for the thylacine: ‘Tasmanian wolf’ or ‘marsupial wolf’ [e.g., 26,27]. Detailed morphological analyses, however, produce contradictory results, with many studies finding little morphological or functional overlap with these traditional convergent analogues [28–33]. For example, bite force estimations suggest a large-prey, hypercarnivorous diet [34], but other biomechanical analyses suggest that the skull was poorly suited to the stress of handling large prey items [35, 36]. Relatively small prey size is also suggested by the energy budget requirement related to the thylacine’s body mass [37]. These contradictory results suggest that the analogous nature of the thylacine and gray wolf/dog complex is not particularly useful from an ecological point of view, and that other analogues should be considered to reconstruct the functional ecology of the thylacine [28].

Here, we address the functional predatory ecology of the thylacine using phenotypically convergent analogues. To place the thylacine into an ecological framework, we recorded the dietary category and preferred prey size of 56 species of faunivorous (animal-consuming) terrestrial mammals, and quantified their cranial shape using Three-Dimensional Geometric Morphometrics (3D GM). Precise convergent analogues were identified using two complementary tests of convergent evolution, the C1–C4 distance-based [38] and the search.conv phenotypic vector angle-based [39] methods. We then used phylogenetic comparative methods to investigate patterns of correlation within cranial shape, dietary category, and prey-to-predator body mass across faunivorous mammals. Using these data, we propose a refined determination of the functional predatory ecology of the thylacine. Our results show that the thylacine is most strongly phenotypically convergent with mid-sized, small prey-focused canids, not the wolf or dog, and most likely preferred prey less than half of their body mass.

## Results

### Shape analysis of the faunivorous cranium

**Fig. 2:**
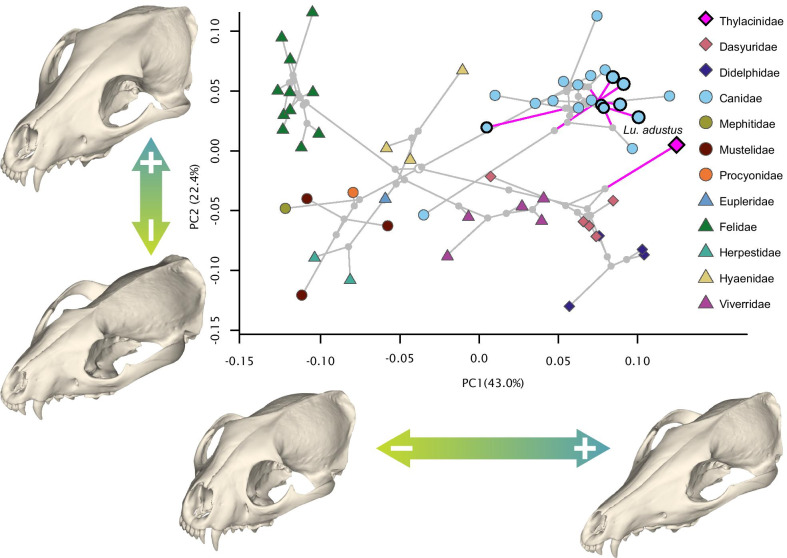
Phylomorphospace of species mean cranial shape for 57 faunivorous mammals. Shape extremes of PC1 and PC2 generated by thin-plate spline warping of the mean skull mesh configuration. Tree root is indicated by open grey node, branches leading to species found to be significantly convergent with the thylacine in the total cranial dataset indicated in magenta and tips circled in black. The convergent species closest to the thylacine in morphospace, *Lupulella adustus*, is labelled

**Fig. 3:**
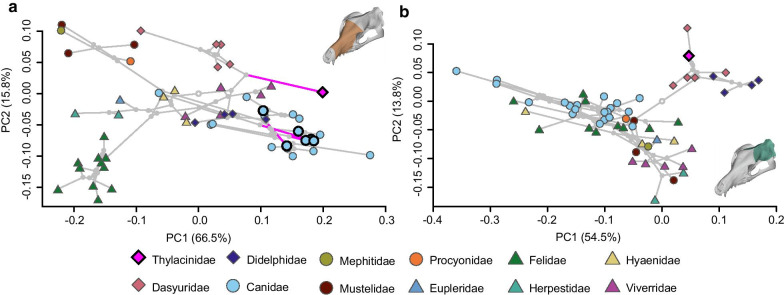
Phylomorphospace of **a** species mean facial shape and **b** neurocranial shape for 57 faunivorous mammals. Tree root is indicated by open grey node, and branches leading to species found to be significantly convergent with the thylacine in each respective dataset (facial or neurocranial) are indicated in magenta and tips circled in black

A projection of the phylogeny into the cranial morphospace (phylomorphospace) generated by the first two Principal Components (PC; 65.4% var.) is shown in (Fig. 2; Additional file 1: Figures S1–2). The general trend is a separation of species with a ‘cat-like’, short, wide rostrum and those with a ‘dog-like’, elongate and narrow rostrum across PC1. The second principal component roughly describes variation in shape related to the degree of dorsoventral compression/inflation of the cranial vault and protraction/retraction of the glenoid of the zygomatic arch, shifting the origin and angle of insertion of the *m. temporalis*. Variance in the morphospace of the facial patch dataset is largely explained by the first principal component (66.5% var.), which again generally describes variation related to a tall, wide ‘cat-like’ rostrum contrasting with an elongate, narrow ‘dog-like’ rostrum (Fig. 3a; Additional file 1: Figures S3–4). The shape extremes of the second component (15.8% var.) describe a relatively low and wide midface with relatively constricted frontals, contrasting with a relatively tall and narrow midface with expanded frontals. Within the neurocranial patch dataset, most of the shape variation is described by the first four principal components (84.5%), with the first two axes accounting for 68.2% of that variation (Fig. 3b; Additional file 1: Figures S5–6). The first component describes a neurocranium with a wide postorbital constriction, short and globular braincase, and diverging temporal lines at the negative extreme. Positively, the extreme represents a narrow postorbital constriction, a subconical and narrow braincase, and temporal lines converging into a sagittal crest. The second component describes a rounded, ellipsoid braincase with a tall and relatively ‘U’-shaped nuchal crest, contrasting with a short and anteriorly-constricted braincase with a low, broad triangular nuchal crest.

**Fig. 4:**
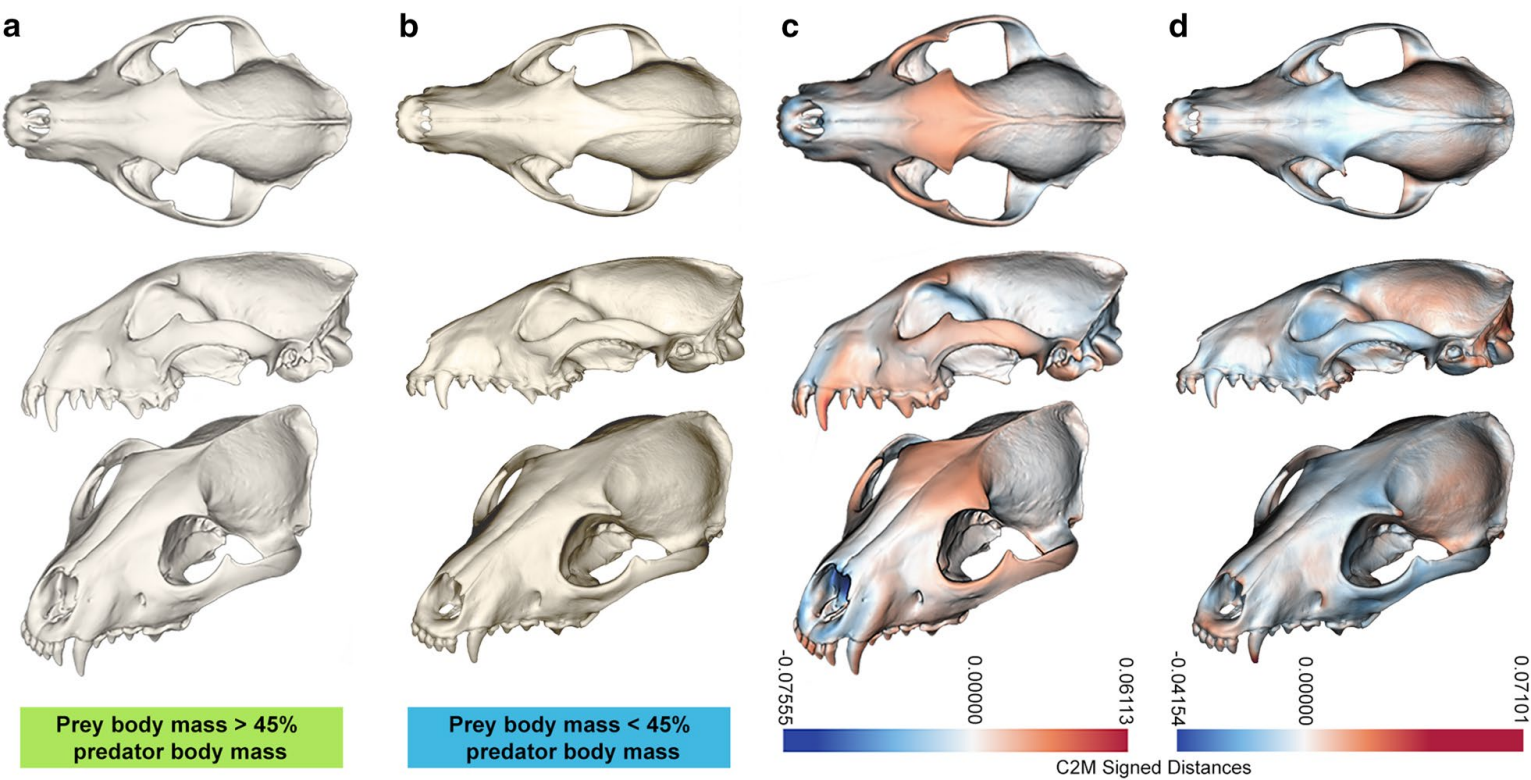
Mean cranial shape of large- and small-prey predators. **a** Large-prey (> 45% of predator body mass) mean shape and **b** small-prey (< 45% of predator mass) mean shape cranial meshes were generated by thin-plate spline warping of the mean cranial shape. Euclidean distances between the meshes of the **c** large-prey and **d** small-prey mean shapes and the total mean cranium shape are shown as deviation from the mean shape expressed by colour (blue–white–red). Blues show constriction relative to the mean shape, reds show expansion, with white as approximately congruent to the mean shape

Cranial shape across placental and marsupial faunivores correlates with prey size, with the prey/predator body mass accounting for 13.0% of the variance in the total cranial dataset [phylogenetic generalised least squares (PGLS) *R*^*2*^ = 0.130, *F* = 8.041, *p* = 0.005; Fig. 4; Table 1]. The multivariate phylogenetic signal (*K*_mult_) is significant but low (*K*_mult_ = 0.133, *p* = 0.001), and there is significant size-related shape variation (evolutionary allometry; PGLS *R*^*2*^ = 0.353, *F* = 29.977, *p* = 0.001). Diet, in either coarse- or fine-grained categories (three or ten dietary categories, see “Methods” below), shows no significant correlation with shape (PGLS coarse: *R*^*2*^ = 0.028, *F* = 0.763, *p* = 0.498; fine: *R*^*2*^ = 0.136, *F* = 0.805, *p* = 0.591). The facial and neurocranial patch datasets both show, without exception, strongly similar trends in significance across the variables (Table 1).

**Table 1:** Canonical Variates Analysis (CVA) prey/predator body mass ratio group discrimination

Module	Prey Size Group Discrimination		
	Total group (*n* = 206)	Placentalia (*n* = 170)	Marsupialia (*n* = 36)
Total (cranium)	87.4%	87.1%	88.9%
Facial	80.1%	78.2%	88.9%
Neurocranial	87.4%	87.1%	88.9%

Percentage of correct relative prey size group attribution within each dataset. Cross-validation is by leave-one-out jack-knife method

### Discrimination of prey size category and the thylacine

Canonical Variate Analyses (CVA) were performed with all PCs accounting for > 1% of variance in each dataset—ten PCs for the whole cranium, seven for the facial subset, and nine for the neurocranium subset. These PCs were tested for the discrimination of relative prey size, using the small- and large-prey categories (< 45% of predator body mass or > 45% of predator body mass) found by the best-fit model of Carbone et al. [40]. Correct total discrimination rates of 80.1–87.4% attained from the datasets, placing predators into small-prey (< 45% predator body mass) and large-prey (> 45% of predator body mass) groups relatively well (Table 2; Additional file 1: Table S1).

**Table 2:** Species found to be maximally convergent with the thylacine

Species	Cranium				Face				Neurocranium			
	C1	*p*	θ	*p*	C1	*p*	θ	*p*	C1	*p*	θ	*p*
*Ch. brachyurus*	0.507	**0.001**	44.4°	**0.006**	0.567	**0.008**	36.2°	**0.004**	0.271	0.062	80.7°	0.073
*Lu. adustus*	0.537	**0.002**	35.4°	**0.001**	0.639	**0.004**	37.6°	**0.009**	0.196	0.143	116.4°	0.201
*Lu. mesomelas*	0.524	**0.001**	46.0°	**0.006**	0.625	**0.005**	39.6°	**0.015**	0.076	0.420	114.6°	0.192
*Ly. gymnocercus*	0.417	**0.006**	51.7°	**0.005**	0.561	**0.013**	37.1°	**0.012**	0.014	0.694	128.1°	0.282
*Ca. latrans*	0.452	**0.003**	41.4°	**0.001**	0.305	0.127	42.6°	**0.020**	0.384	0.015	107.8°	0.171
*Ly. culpaeus*	0.489	**0.004**	38.1°	**0.003**	0.376	0.057	38.4°	**0.008**	0.515	**0.004**	96.4°	0.141
*Vu. vulpes*	0.333	**0.023**	49.3°	**0.006**	0.356	0.076	33.5°	**0.004**	0.189	0.166	125.5°	0.237
*Ca. lupus*	0.201	0.139	43.7°	**0.004**	0.236	0.220	51.3°	**0.032**	0.318	0.043	72.1°	0.051
Dingo	0.182	0.144	50.0°	**0.009**	0.305	0.114	47.2°	**0.020**	0.271	0.082	84.8°	0.091

Species found to be significantly convergent with the thylacine across both phenotypic convergence tests (C1 and search.conv) across the total cranial dataset and the facial or neurocranial patch dataset. Three species (*Ca. latrans*, *Ly. culpaeus*, and *Vu. vulpes*) fail to meet the ad hoc analysis requirements by a single test, but are included here for comparison, along with the commonly-cited ‘convergent’ wolf/dog species complex (represented by *Ca. lupus* and the dingo). C1 values are the scaled phenotypic distances closed between the lineages, θ are the angles between the multivariate phenotypic vectors of the lineages. Bold values indicates significance, adjusted for multiple tests by Benjamini–Hochberg FDR correction, α = 0.10

**Fig. 5:**
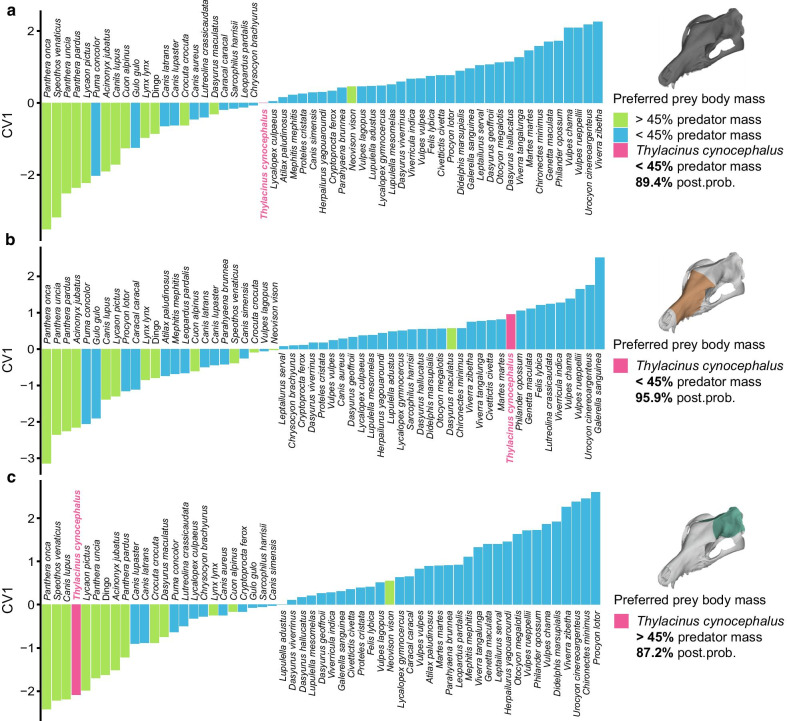
Discrimination of prey size in the thylacine. Canonical Variates Analysis (CVA) discrimination scores of preferred prey size (> 45% or < 45% of predator body mass) within the **a** total cranial, **b** facial, and **c** neurocranial datasets. Group assignment of the thylacine listed to the right of each respective plot, along with the posterior probability (post. prob.) of the assignment

Both the whole cranium and facial patch analyses strongly place the thylacine within the small-prey group (prey < 45% of predator body mass; Fig. 5a, b). The neurocranial patch analysis, however, places the thylacine into the large-prey group (prey > 45% of predator body mass; Fig. 5c). We found that within the total species sampled, PC3 and PC4 are significantly correlated with the prey/predator body mass ratio (PC3 Wilcoxon ranked sum: *p* < 0.001, Spearman’s correlation: r_s_ = 0.476, *p* < 0.001; PC4 Wilcoxon ranked sum: *p* < 0.001, Spearman’s correlation: r_s_ = 0.376, *p* < 0.001; Additional file 1: Table S2). A Spearman’s correlation test just within the placental carnivorans between the PCs and the prey-to-predator mass ratio likewise shows a relatively strong correlation (PC3: r_s_ = 0.500, *p* < 0.001; PC4: r_s_ = 0.454, *p* < 0.001). The Spearman’s test within the marsupial species, however, fails to find a correlation between the PCs and the prey-to-predator mass ratio (PC3: r_s_ = 0.070, *p* = 0.683; PC4: r_s_ = 0.196, *p* = 0.253), though this result may be an artefact of the small marsupial sample size and should be viewed with caution.

### Convergence with the thylacine

**Fig. 6:**
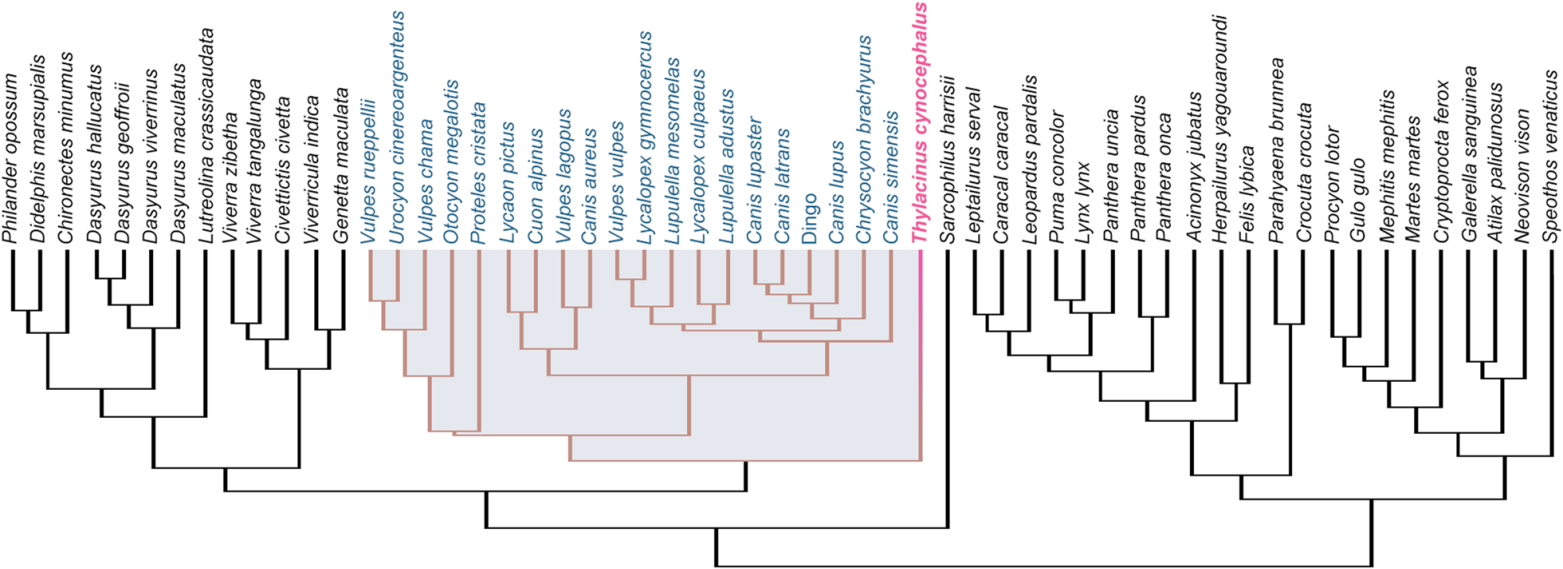
Candidate convergent species cluster phenogram. Unweighted Pair Group Method with Arithmetic mean (UPGMA) phenetic cluster analysis on the PC scores accounting for > 1% variance within the total cranial dataset. This group of species forms the candidate species input for all convergence analyses. The thylacine and the phenetic candidate group are called out in colour

Species grouping with the thylacine in an Unweighted Pair Group Method with Arithmetic mean (UPGMA) hierarchical cluster analysis using mean PC scores accounting for > 1% of shape variance were selected as candidates for phenotypic convergence (Fig. 6). These candidate species were then pairwise tested for convergence with the thylacine using both distance-based C1–C4 [38] and multivariate phenotypic angle-based [39] methods (see “Methods” section below for details). Candidates that were found to be significantly convergent by both methods (i.e., both significant C1 and significant phenotypic angle) across the total cranial and either the facial or neurocranial datasets were considered to be maximally phenotypically convergent species with the thylacine.

The group of maximally convergent species, i.e., those that were found to be significantly convergent with the thylacine in two of the three datasets under both methodologies, is comprised of four canids: *Chrysocyon brachyurus* (maned wolf), *Lupulella adustus* (side-striped jackal), *Lupulella mesomelas* (black-backed jackal), and *Lycalopex gymnocercus* (Pampas fox; Table 3). Three species of note that missed the ad hoc significance cut-off by one analysis were *Canis latrans* (coyote), *Vulpes vulpes* (red fox), and *Lycalopex culpaeus* (culpeo)*.* Other species that have classically been noted as convergent analogues with the thylacine, i.e., *Canis lupus/*dingo, are found to have less support (Table 3; see Additional file 1: Tables S3–5).

**Table 3:** PGLS and phylogenetic signal results

	Total cranium dataset K_mult_ = 0.1328, *p* = **0.001**					Facial patch dataset K_mult_ = 0.1724, *p* = **0.001**				Neurocranial patch dataset K_mult_ = 0.1181, *p* = **0.001**			
Function	Df	R^2^	F	Z	*p*	R^2^	F	Z	*p*	R^2^	F	Z	*p*
Pcoords ~ ln(Csize)	1.55	0.353	29.977	4.839	**0.001**	0.262	19.521	3.905	**0.001**	0.443	43.701	4.887	**0.001**
Pcoords ~ clade	2.54	0.003	0.069	− 4.680	1.000	0.003	0.072	− 4.187	1.000	0.003	0.077	− 3.893	1.000
Pcoords ~ prey/predator mass ratio	1.54	0.130	8.041	3.214	**0.005**	0.094	5.577	2.434	**0.007**	0.088	5.205	2.432	**0.014**
Pcoords ~ dietary category (coarse)	2.53	0.028	0.763	− 0.084	0.498	0.016	0.441	− 0.874	0.800	0.077	2.214	1.514	0.087
Pcoords ~ dietary category (fine)	9.46	0.136	0.805	− 0.287	0.591	0.124	0.721	− 0.592	0.721	0.208	1.339	0.781	0.217
ln(Csize) ~ clade	2.54	0.001	0.023	− 2.409	0.973	0.001	0.023	− 2.307	0.969	0.001	0.031	− 2.189	0.968
ln(Csize) ~ prey/predator mass ratio	1.54	0.111	6.749	1.467	**0.019**	0.089	5.294	1.373	**0.029**	0.116	7.077	1.515	**0.019**
ln(Csize) ~ dietary category (coarse)	2.53	0.084	2.432	1.163	0.106	0.091	2.655	1.182	0.080	0.087	2.528	1.167	0.105
ln(Csize) ~ dietary category (fine)	9.46	0.175	1.086	0.366	0.354	0.198	1.263	0.567	0.288	0.183	1.146	0.437	0.329

Multivariate phylogenetic signal test (K_mult_) results indicate low but significant signal in each dataset. All significant results after Benjamini–Hochberg adjustment (α = 0.10) in bold

Distance-based (C1; see “Methods”) pairwise comparisons indicate that *Lu. adustus*, *Lu. mesomelas*, and *Ch. brachyurus* all show greater than 50% convergence with the thylacine in both the whole cranium and facial analysis (Table 3; Additional file 1: Tables S3–5). Within the neurocranial patch subset, only *Ly. culpaeus* shows a significant result, closing just over 50% of phenotypic space with the thylacine, though this result is not found by the phenotypic angle method (Additional file 1: Table S5)*.* Considering the commonly posited convergent species in the total cranium analysis (*Ca. lupus, V. vulpes,* and the dingo), only *V. vulpes* shows a significant (~ 33%) convergence with the thylacine. None of those three species show significant levels of convergence in the analyses of the facial patch or neurocranial patch subsets.

A roughly similar pattern of convergence is indicated by the search.conv phenotypic angle method (Table 3; Additional file 1: Tables S3–5). The majority of the larger-bodied (> 5 kg) canids all show significantly small angles within the total cranium analysis, ranging from 76.1° in *Cuon alpinus* (dhole) to 35.4° in *Lu. adustus.* The hypercarnivorous *Cu. alpinus* and *Lycaon pictus* (painted wolf) are the only canids tested not to show a significantly small phenotypic angle in the facial subset, with the remaining canids ranging from 51.3° in *Ca. lupus* to 33.5° in *V. vulpes.* The search.conv analysis fails to locate any significantly small angles within the neurocranial dataset.

## Discussion

### Morphological convergence and the thylacine

**Fig. 7:**
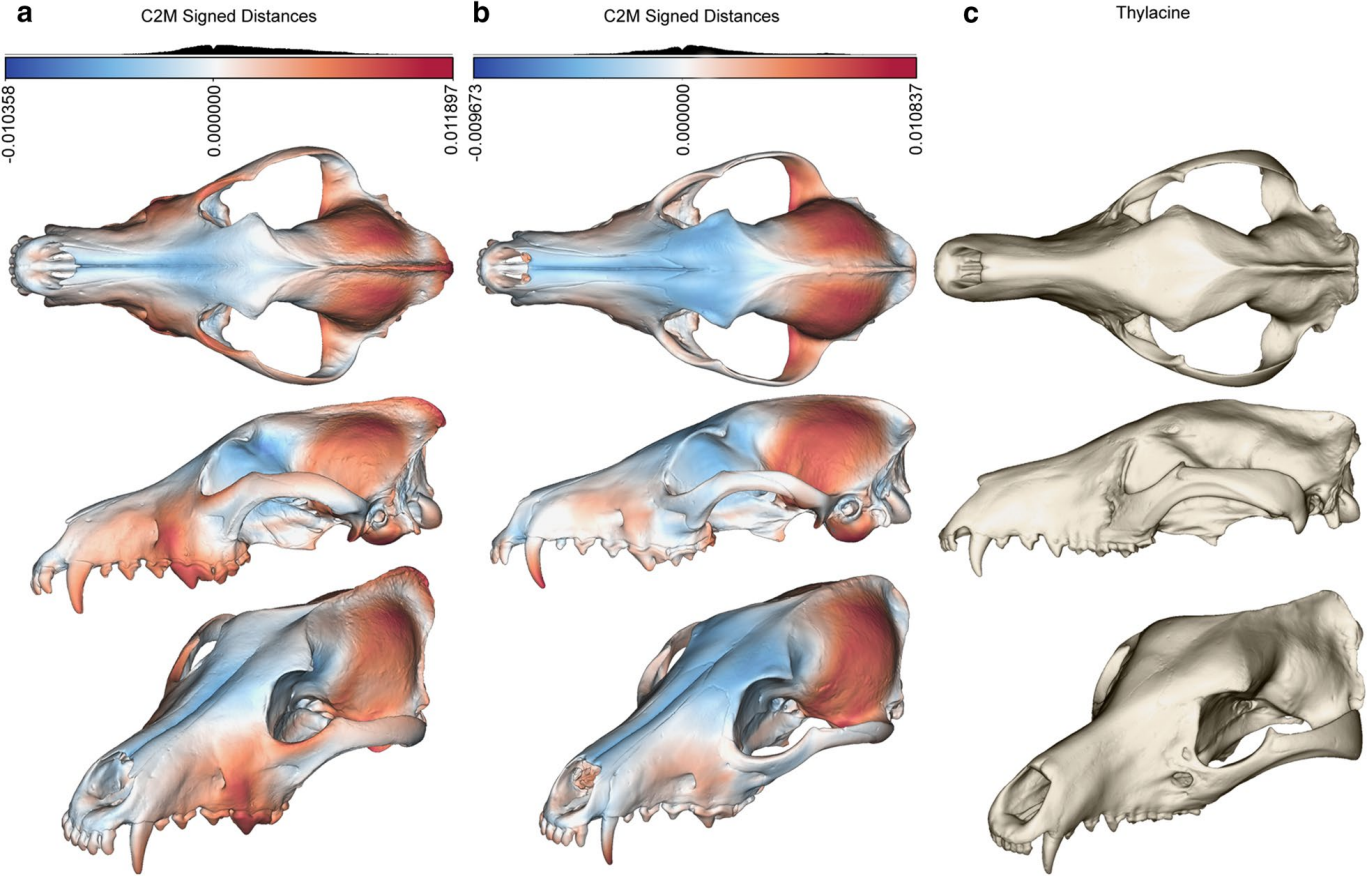
Comparison of cranial shape between the thylacine, wolf, and maximally convergent species group. **a** Gray wolf (*Canis lupus*) mean cranial shape and **b** maximally convergent group (*Chrysocyon brachyurus, Lupulella adustus, Lu. mesomelas, Lycalopex gymnocercus*) mean cranial shape, with the Euclidean distances from the **c** mean thylacine shape shown by colour (blue–white–red), as in Fig. 4 above

Previous studies have viewed the thylacine from a starting point of convergence with the gray wolf/dog species complex [19–25]. We find little support for morphological convergence of the thylacine cranium within these species. Rather, we find repeated and substantial support for convergence with a specific group of canids, the African jackals and South American ‘foxes’, that share a distinct feeding ecology separate from that of the gray wolf/dog species complex (*Canis lupus *sensu* amplo*) (Fig. 7). These convergent species are, broadly speaking, mid-sized (5–25 kg) carnivores with an average prey size < 45% of their own body mass. Our results show little to suggest that the thylacine was morphologically convergent with the gray wolf/dog species complex, and by extension little to suggest ecological similarity.

Outside of the wolf/dox complex, the thylacine has previously been suggested to be morphologically similar to *V. vulpes* [24, 28], and phenotypic convergence between the two species has previously been assessed and interpreted as highly significant [41]. While we do find some support for convergence with *V. vulpes*, echoing these previous findings, we find much stronger repeated support outside of the true fox group. The previous phenotypic convergence study [41] fundamentally differed in purpose and design from the current study, and was tailored to assessing a concept of general convergence with canids within a broad grouping of mammals, not with the intent to identify ecological analogues. All aspects of that study design reflect this, from the taxa selection (inclusive of non-ecologically-meaningful species, e.g., koalas and wallabies) to the source of the landmark protocol/dataset [42], which avoided functionally-relevant areas of the cranium, such as the rostrum. Although that study was not designed to find informative convergent analogues with nor to infer the ecology of the thylacine, it did find that the thylacine was closer in phenotype to canids than expected from phylogeny [41]. That being said, the study was not attempting to, and did not, show with any precision which canids were most like the thylacine, why they were, or what that similarity may mean functionally or ecologically.

### Prey size, morphology, and the thylacine

Cranial and facial shape place the thylacine with other predators that routinely take prey < 45% their own body mass, based on both morphospace occupation and canonical variate discrimination. This supports research showing that the cranium and mandible of the thylacine would perform poorly under the stresses encountered in taking large-bodied prey [35, 36, 43]. This is also consistent with interpretations following recent estimates of thylacine average body mass at ~ 16.7 kg [37]. Most mammalian predators take prey substantially smaller than themselves, in part due to the energy expenditure vs. intake costs brought about by locating, capturing, and killing generally uncooperative prey [40, 44–46]. This is especially true for carnivores under 21 kg in body mass, where foraging costs do not outweigh the metabolic demands of the predator, and are less than the costs and associated dangers of capturing and killing large-bodied prey. Within large-bodied predators over 21 kg (e.g., *Canis lupus*), there is a tendency to switch to prey larger than 45% of their own mass, due to the need to increase the net gain per hunting effort as their absolute metabolic rate scales with body mass—it becomes too costly to find, catch, and consume enough small meals, so they tend to switch to larger, and more difficult, prey.

Despite the thylacine being commonly considered as a ‘marsupial wolf’ [e.g., 27,47,48], some authors have been sceptical regarding such predatory capabilities in the thylacine [19, 29]. Two general strategies for procuring large and potentially dangerous prey are seen in extant mammalian carnivores. Felids tend to be ambush hunters, with powerful forelimbs capable of supination for the capture and restraint of prey, and robust, shortened rostra to deliver crushing or locking bites to the head, muzzle, and neck. Large hypercarnivorous canids (e.g., *Ca. lupus, Cu. alpinus*, and *Lycaon pictus*) tend to be highly social group-living pursuit hunters. These canids possess elongate and reduced distal limbs enabling efficient locomotion and use their pack size to overcome large prey with numerous shallow or tearing bites. The cranium of the thylacine is not cat-like, and the elongate and narrow rostrum precludes the muzzle, throat, or nape/back of skull bite used by large felids. Additionally, there is little indication of locomotor specialisation in the postcranial skeleton, and studies have shown that neither the limb proportions nor the forelimb morphology support a specialised, cursorial habit, nor do they support the ambush of large-prey [31–33]. While the thylacine may share similar cranial morphology to the African jackals and South American ‘foxes’, it shows none of the specialised limb morphology that these canids possess, which all show varying degrees of the cursorial specialisations (e.g., limb elongation, distal element reduction and compression) shared by *Ca. lupus*. Rather, the forelimb of the thylacine seems to be that of a relatively generalised ambush or pounce predator lacking the anatomical specialisations required to handle large prey.

The morphospace occupation and canonical variate discrimination results are echoed in the feeding habits of the canids found to be strongly convergent with the thylacine. This group of canids (*Ch. brachyurus, Lu. adustus, Lu. mesomelas,* and *Ly. gymnocercus*) as a whole focuses on prey far below their own body mass, mostly small vertebrates such as rodents and lagomorphs. Two species recovered as significantly convergent in both total cranial analyses but not recovered as such across both facial patch analyses are the ~ 6 kg V*. vulpes* and the ~ 15 kg *Ca. latrans.* Like the above canids, the diet of the red fox is also comprised largely of rodents, though it is a flexible and opportunistic predator that will occasionally take small mammals up to ~ 3.5 kg, roughly 50% of their body mass [49–51]. The coyote primarily consumes roughly similar-sized prey to the above canids, with lagomorphs making up the majority of its diet across much of its range. However, *Ca. latrans* has a highly flexible social structure, and in packs are capable of predation on relatively large-bodied prey, such as juvenile cervids [51–53]. The ~ 9 kg *Ly. culpaeus*, found to be significantly convergent with the thylacine in the neurocranial dataset only by the distance-based analysis, also has a dietary regime roughly similar to that of the red fox and coyote. Considered the most carnivorous of the South American ‘foxes’, the diet of *Ly. culpaeus* comprised mostly of rodent and lagomorph prey, though it is noted to often prey on the largest of the small mammals available, e.g., hares and occasionally newborn–juvenile domestic sheep [51, 54]. None of these three small prey-focused canids that are able to take larger-bodied prey (the culpeo, red fox, and coyote) are found to be significantly convergent with the thylacine across both facial patch analyses, with the culpeo not found to be significantly convergent in either facial patch analyses.

The neurocranial patch CVA grouping of the thylacine with large-prey specialists echoes a similar result found in marsupials by using muscle cross-sectional area to estimate bite force [34]. Within that study, the estimated bite forces for marsupials, and the thylacine in particular, were found to be exceptionally high, both relatively and absolutely. These results are not supported by biomechanical analyses of the thylacine cranium, which find it particularly unsuited to handle the stress of either producing such high bite forces or of handling large prey items, nor by the feeding ecology of some of the marsupials examined [34–36, 43]. The cross-sectional area available for muscle tissue is negatively affected by brain expansion, which limits the area available for musculature between the neurocranium and zygomatic arch. Marsupial carnivores have endocranial volumes that are approximately 40% of the volume in a placental carnivore of similar body mass, creating a much larger cross-sectional muscle area available for a given body size, and seemingly regardless of average prey size [34, 55]. We find that while 3D neurocranial shape does correlate with prey size in placental carnivores, it does not seem to be strongly correlated with prey size within marsupials, though the small sample size prevents any firm conclusion. Cross-sectional muscle area, and by extension neurocranial shape, may not be a good predictor of in vivo bite force in marsupials, as previously noted [35], and may not correlate with prey size in marsupials.

### Morphology and diet

Surprisingly, we find no correlation between diet and cranial shape, a result in contrast to that of previous studies [e.g., 21,42]. This is possibly due to our focus on carnivorous species; we avoided including herbivorous carnivorans and those trending towards frugivory or omnivory, restricting the phenotypic range. Furthermore, relative size of the food object consumed may be a larger constraint on the cranium of faunivores than the material properties of the food [e.g., see 56]. A diet-based pattern might emerge if we included more disparate, herbivorous species, or sampled data from the dentition, as it actively engages with the food [57].

### Concepts of convergence

The concept of phenotypic convergence is necessarily broad, and itself has many different possible interpretations. As a classic example of convergence, the ichthyosaurs are noted to have strongly converged on the same general body plan as fish [58], and the degree of convergence between thunnosaurian ichthyosaurs and lamnid sharks is striking [59]. However, ichthyosaurs displayed a range of body plans [60], from eel-like to tuna-like, and fish themselves display an incredibly vast array of body plans and ecologies. The issue then becomes, like so many others in science, one of scale or resolution: at what level are you invoking the concept of convergence? It is true that ichthyosaurs were convergent with fish, and that some were convergent with lamnid sharks. However, it is equally true that those ichthyosaurs were not convergent with all fish, and that some were not convergent with lamnid sharks. If meaningful inferences about the functional ecology of an extinct animal is the intent of the convergence study, then the data (comparative taxa, phenotype, etc.) should also meaningfully reflect the question.

When trying to understand the functional ecology of an extinct animal by comparison with modern analogues, broad scale concepts of convergence can be helpful and evocative. Such examples of broad scale convergence as the ichthyosaurs above, or as between ceratopsians and bovids [61], crocodilians and odontocetes [62], borophagine canids and hyaenids [63], or toxodontids and hippopotamids [64] can offer support for broad ecological similarities. However, this broad scale interpretation of convergence can fail to recover a higher fidelity view of the functional ecology of an extinct animal, since bovids, odontocetes, hyaenids, canids etc., each encompass animals with widely disparate ecologies. By framing both the concept of convergence and interpreting the resulting data within a high-resolution functional ecology study design, we can start to form much more precise hypotheses of the ecologies of extinct animals.

This is not to say, however, that a precise one-to-one matching between extinct and extant comparatives is necessarily the goal of such a study, or even possible. The thylacine here does not actually fall within the morphospace of any living canid comparatives, strongly suggesting that it is not directly comparable with any of these canids, whether jackal or wolf (Fig. 4a–c). A lack of direct correspondence here is unsurprising, as many previous studies have noted that the phenotypic similarities are largely superficial [31–33], and a concept of the thylacine as strongly ecologically convergent with African jackals is probably rather wrong. But, when trying to reconstruct the functional ecology of an extinct animal it is better to be *less* wrong, and identifying more precise analogues supports better and more informative reconstructions.

## Conclusion

We find little support for morphological convergence of the thylacine with the gray wolf/dog species complex. Our data instead show strong support for a morphology similar to the African jackals and South American ‘foxes’, and slightly lesser support for similarly to the coyote and red fox. Similarly, we find no support for the thylacine cranium to be suited for the handling of large-bodied prey, and instead find that it comfortably groups with other predators that routinely take prey < 45% of their own body mass. Taken together with the recent revision of the average thylacine body mass [37], these findings reconcile previous contradictory morphological studies of thylacine predatory ecology. This work highlights that extending concepts of convergence beyond superficial similarity can provide much more precise interpretations of the functional ecology of extinct animals, and that broader comparisons can lead to poor or uninformative interpretations. We suggest that the thylacine is likely to have been ecologically most similar to African jackals or South American ‘foxes’ in terms of feeding habit (though probably not hunting strategy), likely specialising on the peramelemorphians and smaller macropodids present throughout its prehistoric and historic range.

## Methods

### Specimens and phylogeny

**Fig. 8:**
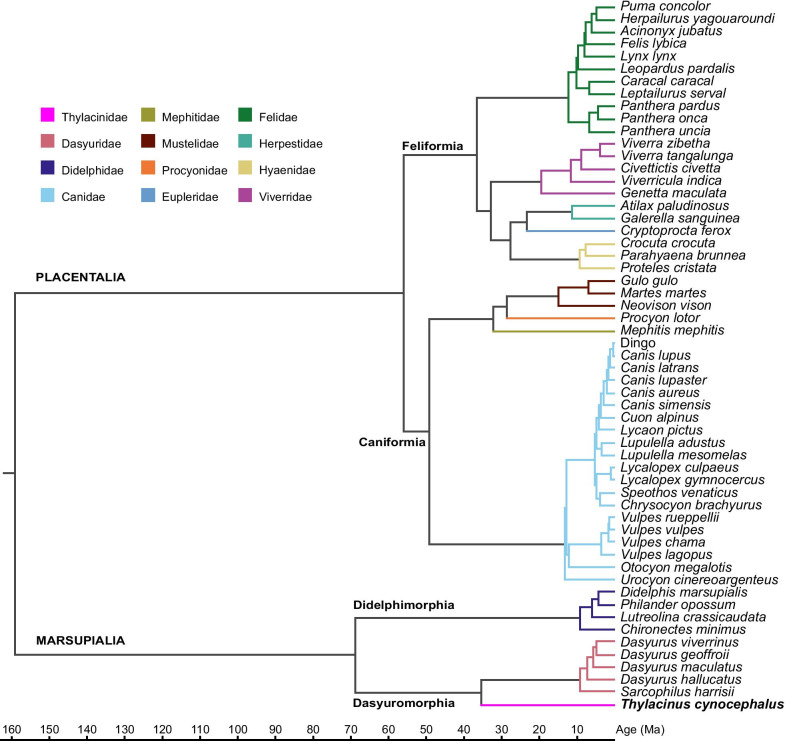
Time-scaled phylogeny of faunivorous mammals used in this study. Assembled using topologies and divergence dates from recent studies (see Additional file 1: Table S7)

We sourced specimens from 13 institutions: American Museum of Natural History (New York, USA), Australian Museum (Sydney, Australia), Ditsong National Museum of Natural History (Pretoria, South Africa), Michigan State University (Lansing, USA), National Museums Victoria (Melbourne, Australia), Natural History Museum, Berlin (Germany), Natural History Museum, London (UK), Smithsonian National Museum of Natural History (Washington, D.C., USA), South Australian Museum (Adelaide, Australia), State Museum of Natural History, Stuttgart (Germany), Tasmanian Museum and Art Gallery (Hobart, Australia), University Museum of Zoology (Cambridge, UK), and Western Australian Museum (Perth, Australia). A total of 223 specimens across 57 faunivorous species were sampled from Carnivora and Marsupialia, representing nine carnivoran and three marsupial families (Fig. 8, Additional file 1: Table S6). Comparative selection was based on previous hypotheses of convergence, as well as recorded diet and body mass to minimize allometric and ecological biases. Preference was given to more carnivorous members of lineages, due to the derived shearing carnassial complex of the thylacine indicating a hypercarnivorous diet [consisting of > 70% vertebrate flesh; 11, 30, 65]. Additionally, extreme body sizes were avoided, sampling between ~ 1–80 kg. However, since the thylacine’s closest extant relatives are the relatively small dasyurids (0.07–10 kg) some smaller carnivoran and didelphid species were included to reflect the body sizes of the dasyurids included in the study. As the thylacine is often considered convergent with canids, we included 20 species of canid representing the three extant clades (Canina, Cerdocyonina, and Vulpini) and the sister taxon *Urocyon cinereoargenteus* (gray fox). All specimens were adult, classified by full dental eruption and occlusion, and in carnivorans the fusion of the basisphenoid/basioccipital suture. Where possible, four specimens (two female, two male) of each species were sampled with the exception of the thylacine (*n* = 16; 7 female, 7 male, 2 unknown sex). An informal, time-scaled, composite phylogeny was assembled in Mesquite v3.6 [66] using topologies and divergence dates from recent studies (Additional file 1: Table S7). This tree was used in all subsequent phylogenetically-informed analyses.

### Shape analysis (geometric morphometrics)

Specimens were either surfaced scanned with an Artec Spider/Space Spider structured light scanner or Computed Tomography (CT) scanned, with 3D polygon mesh models produced in Artec Studio 12 (Artec Group, Luxembourg) or Avizo 7 (Thermo Fisher Scientific), respectively. Specimens with small amounts of unilateral damage were imported into Geomagic Studio 2014 (3D Systems, USA) and restored using the mesh editing tools to allow for bilateral landmark placement; additional vertex cleaning was performed in MeshLab v2016.12 [67] prior to 3D landmarking.

**Fig. 9:**
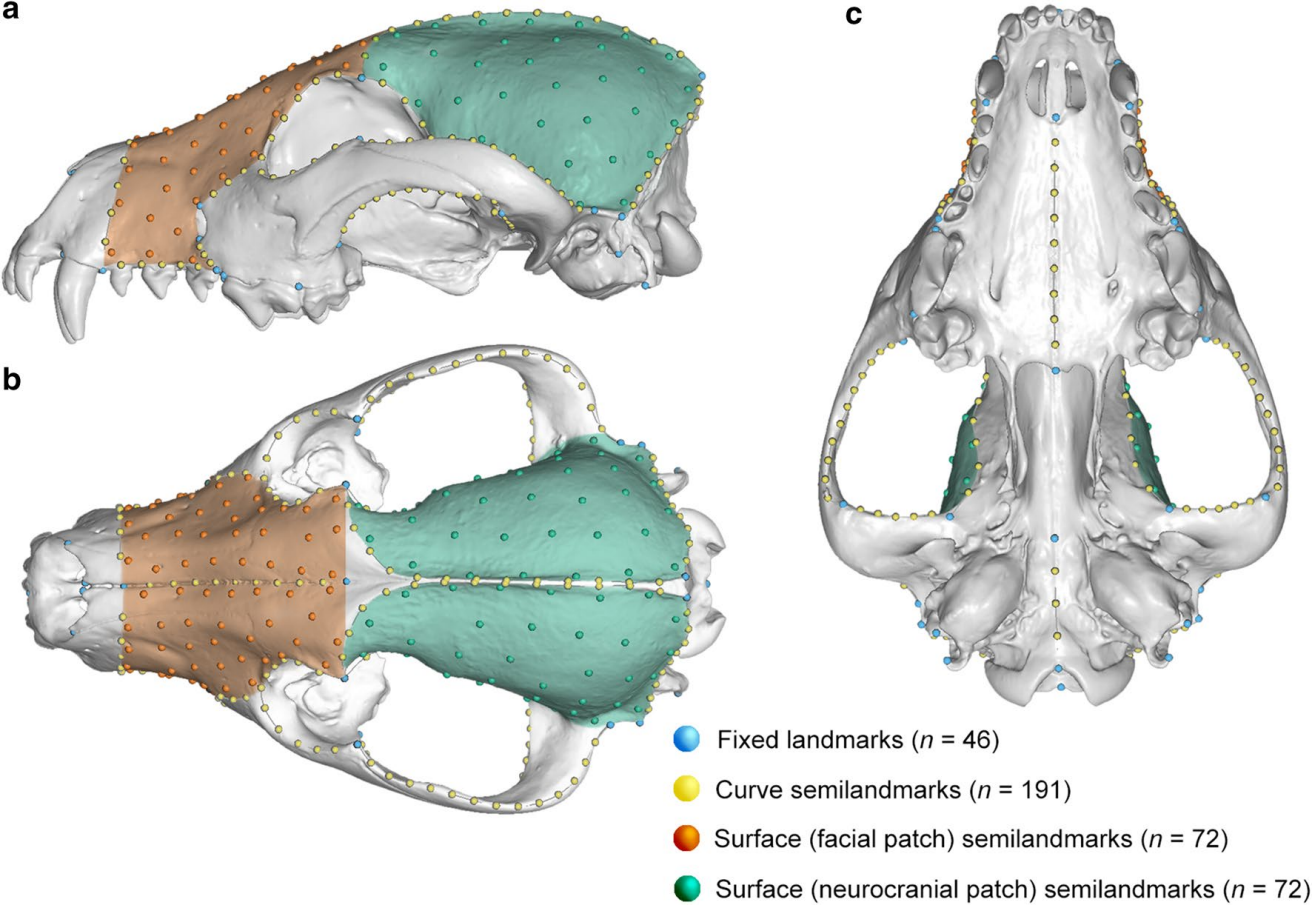
Landmark and semilandmark template. Template created on the approximately mean shape cranium of *Cuon alpinus* (dhole), shown in **a** lateral, **b** dorsal, and **c** ventral views. For definitions and detailed protocol, see Additional file 1: Table S8

To capture functionally relevant shape data, a set of 381 landmarks (46 fixed landmarks, and 191 curve and 144 patch semilandmarks) was established on the scanned cranial specimens (Fig. 9; see Additional file 1: Table S8 for definitions and detailed protocol). Landmarking of all specimens was performed by DSR in Viewbox 4 (dHAL software, Greece). Coverage was emphasised on the ecologically relevant areas of the rostrum and neurocranium/origin of the muscles of mastication. These point coordinates were then exported in three different sets: the total data set (*n* = 381), the facial patch (*n* = 72), and the neurocranial patch (*n* = 72). Dividing the face and neurocranium allows for the separate interrogation of these functional modules [68, 69] in addition to the analysis of the total dataset, which is especially relevant considering the markedly smaller endocranial volume (and thus different neurocranial shape) seen in some marsupials [55, 70, 71]. These landmark coordinates were each subjected to Procrustes superimposition to remove translation, rotation, and scaling in the R package geomorph v3.1.3 [72]. The resulting sets of Procrustes coordinates (Pcoords) were used as shape variables for all subsequent analyses below.

### Ecology

Ecological information including diet, average body mass, and average prey body mass was sourced from the literature (Additional file 1: Data S9). Diet was categorised based on the categorisation scheme of Pineda-Munoz & Alroy [73], and recorded as percentage biomass in order to obtain the most important food source, with stomach content analyses preferred, though with a small number of species only scat data were available. The base dietary dataset [73] is heavily biased toward Rodentia, so we formed several new dietary categories for faunivorous species following their categorisation method of main (> 50%) and secondary (20–50%) food sources. This resulted in 10 dietary categories present across our sampled species (carnivore/frugivore, carnivore/insectivore, carnivore, durophage, frugivore, generalist, insectivore/carnivore, insectivore, insectivore/frugivore, and insectivore/herbivore). We also created a reduced set of three diet categories to avoid the potential of overfitting: carnivorous (diet consists of ≥ 50% vertebrates), insectivorous (diet consists of ≥ 50% invertebrates), and generalist (no diet category ≥ 50%). These two methods provided a fine-grained (10-category) and coarse-grained (3-category) set of dietary categories for analyses. Where possible the body mass of species with large latitudinal ranges were sampled and averaged over the geographic range to avoid locale biases. Average prey size was calculated from the dietary sources, again averaged across studies and ranges for species with extensive ranges extending over disparate environments. These average masses were transformed into a prey/predator mass ratio. The values for the pack-hunting predators *Crocuta crocuta* (spotted hyena), *Cu. alpinus*, *Lycaon pictus*, and *Ca. lupus* were divided by average pack size as recorded in the literature in order to remove the potential effect of social behaviour obscuring morphological trends. These prey/predator mass ratios were natural log transformed for all subsequent analyses.

### Statistical analyses

Statistical analyses were performed separately on three data subsets: the total cranial landmark and semilandmark subset, the facial patch semilandmark subset, and the neurocranial patch semilandmark subset. Exploration of shape variation was performed by warping a reference skull mesh to the mean shape of the total cranial dataset using warpRefMesh in geomorph. This was followed by warping the mean mesh shape to each of the principal component axis extremes using the plotRefToTarget function in geomorph. Mean cranial shapes of the large (> 45% of predator body mass) and small (< 45% of predator body mass) prey groups were similarly generated. The species mean PC scores were individually mapped to the phylogeny using the contMap function in the R package phytools v0.6-99 [74]. Phylomorphospaces were also generated by projecting the phylogeny into scatterplots of the species mean PC scores via the geomorph function phylomorphospace.

The level and significance of a phylogenetic signal was tested on the species mean Pcoords using the multivariate K_mult_ statistic via physignal in geomorph. To characterise the relationship of size on cranial shape, we performed a PGLS analysis of species mean natural log-transformed centroid size (lnCS) on shape using the function procD.pgls in geomorph. A further set of PGLS analyses were performed, with the thylacine removed as an unknown case, investigating the influence of dietary category, both fine- and coarse-grained, and prey/predator mass ratios on cranial shape. Ecological variables found to be significantly correlated with shape were then tested against the PC scores accounting for > 5% of variation via Wilcoxon ranked sum tests. Phylogenetically-informed analyses (e.g., phylogenetic ANOVAs) were not used on the PC scores, as PC scores have been noted to potentially be misleading when used in a phylogenetically comparative method [75]. While the Wilcoxon ranked sum test assumes independence of data that cannot be met under a phylogenetic context, it is a relatively robust and forgiving non-parametric test. Nevertheless, the results should be interpreted with this caveat in mind.

Determination of the probable prey size of the thylacine was performed by canonical variate analyses on the PC scores. To minimise the potential for spurious group allocation by using PC axes in a CVA [76], only those PC axes describing > 1% of shape variance of each of the three datasets were used, resulting in 10, seven, and nine PCs used for the total cranial, facial, and neurocranial datasets respectively. We grouped the prey/predator mass ratios into small (prey < 45% of predator mass) and large (prey > 45% of predator mass) categories for the CVA, following the relative prey to predator mass value found to maximise the energetic constraint model fit of Carbone et al. [40, 44]. The analyses were first run on the datasets with the thylacine removed as an unknown variable to generate discriminant functions to best separate the groupings. The resultant discriminant functions were then used to assign group membership to the thylacine, based on the thylacine specimens’ PC scores. As no significant correlation between shape and diet was found after adjusting for phylogenetic relatedness, the dietary categories were not subjected to a CVA.

An Unweighted Pair Group Method with Arithmetic mean (UPGMA) hierarchical cluster analysis was performed using hclust in R on the species mean PC scores accounting for > 1% of shape variance to generate a set of candidate species to test for convergence with the thylacine. This cluster analysis was performed on the total skull dataset to create a generalised grouping of shape to approximate the idea of superficial phenotypic likeness and/or convergence. The resultant group of 20 phenotypically similar species was then tested for convergence with the thylacine in the total skull, facial patch, and neurocranial patch subsets of data.

Convergence was tested by two different methods: the C1–C4 distance-based method [38] in the R package convevol [77] and the conv.search [39] angle of phenotypic vectors method in the R package RRphylo [78]. The C1–C4 method quantifies the Euclidean distance between two tips relative to the maximal distance between nodes within their lineages, producing values representing the phenotypic “distance closed” by convergence between phylogenetic tips. The C1 [1 − (D_tip_/D_max_)] metric calculates the scaled distance between tips and the maximal distance between the two lineages, ranging from 0 at as different as the lineages ever were, to 1 at complete lineage convergence. The overall unscaled magnitude of convergence is given by C2 as the difference between the maximal lineage distance and the distance between the tips (D_max _– D_tip_). C3 and C4 again offer scaled values, with C3 the percentage of evolution accounted by the focal tips relative to that of the total lineage (C2/L_tot.lineage_) whereas C4 provides the proportion of evolution relative to the total clade containing the focal tips (C2/L_tot.clade_). Significance is tested by multiple simulations of evolution under a Brownian motion model along the phylogeny using a variance–covariance matrix derived from the input data, creating an expected distribution of distance measures. This expected distribution is used to assess significance in the measured distributions. It is important to note that the C1–C4 method does not include divergence time within the analyses. Rather, the method only takes into account the magnitude of phenotypic distance between the tree tips relative to that of internal nodes in the phylomorphospace generated by the data.

To include the potential effect of evolutionary time on phenotypic convergence, we used the search.conv method, which calculates the angle (θ) between the multivariate phenotypic vectors of a pair of species. Adjusting for phylogenetic distance is performed by dividing this angle θ by the sum of the branch lengths between the species and their most recent common ancestor. An angle θ of ~ 90° indicates phenotypic dissimilarity. Angles trending towards 180° indicate increasingly opposite phenotypic vectors, with angles trending towards 0˚ indicating increasing phenotypic similarity. Phenotypic similarity of species under a Brownian motion model of evolution is expected to decrease proportionally with increasing time since divergence. Species evolving under a convergent regime should show a more similar phenotype, and thus a smaller angle θ, than expected given their temporal distance. This can be calculated as the mean angle θ for entire clades, to test for convergence between clades, or for disparate groups of species evolving under a putative convergent ‘state’. This latter method can also be used to test for convergence between a focal pair of candidate species by calculating the angle θ between them, e.g., between individual members of the UPGMA-clustered group and the thylacine. To assess significance, the measured angle θ was tested against 1000 angles θ generated by shuffling the convergent ‘state’ randomly across the tips of the given tree.

Candidate species found by UPGMA clustering were pairwise tested with the thylacine using species mean PC scores accounting for > 1% of variance. Distance-based (C1–C4) tests were performed using the function convratsig in the package convevol with 1000 simulations of evolution via Brownian motion, and phenotypic angle θ tests performed using the function search.conv in the package RRphylo, using the ‘state-based’ search. Both of these convergence tests were run on the total skull, facial patch, and neurocranial patch subsets of data. To ease integration of the two methods, only the scaled C1 metric is focused on here, but all results are given in (Additional file 1: Tables S15–17). Species found to be significantly convergent with the thylacine in the total cranium dataset and at least one of the two patch subsets (i.e., facial patch, and/or neurocranial patch) in both the C1 distance-based and search.conv phenotypic angle methods were considered as showing strong support for convergence with the thylacine.

Shape differences between selected warped meshes generated as above were visualised using CloudCompare v2.11.3 [79]. Meshes were registered and scale-adjusted against a reference mesh (either the mean cranial shape, or the mean thylacine cranial shape). These registered meshes were then nearest-neighbour distance compared via the “Compute cloud/mesh distance”, with the signed Euclidean distances between meshes displayed as a colour scalar field. This was performed for the large (> 45% predator mass) and small (< 45% predator mass) prey group mean shapes and the total group mean shape, and between the mean *Canis lupus*, mean C1/search.conv identified convergent group, and the mean thylacine cranial shapes.

All analyses were performed in R v.3.6.1 [80]. Significance results for all analyses using multiple tests were adjusted to a Type I error false discovery rate of 0.10 using the Benjamini–Hochberg procedure [81].

## Supplementary Information

**Additional file 1:** Consists of all supplementary figures and tables cited in the manuscript, and supplementary references for the diet, body mass, prey size data, and phylogenetic tree (found at: https://figshare.com/projects/Convergence_between_the_thylacine_and_small_prey-focused_canids/84905).**Figs S1–6**. PC scores 1–4 mapped onto phylogeny for the total cranial, facial patch, and neurocranial patch datasets.**Table S1.** CVA discrimination coefficients. **Table S2.** Spearman correlation and Wilcoxon ranked sum results. **TablesS3–5.** Results of the C1–C4 and phenotypic angle θ convergence tests for the total cranial, facial patch, and neurocranial patch datasets. **Table S6.** List of specimens, and Morphosource DOI for each surface mesh file. **Table S7.** References for composite phylogenetic tree. **Table S8.** 3D GM landmark definitions and protocol. **Table S9.** References for body mass and diet.

## Data Availability

The datasets and code supporting this study are available at figshare: https://figshare.com/projects/Convergence_between_the_thylacine_and_small_prey-focused_canids/84905. Surface meshes are available for download at Morphosource, subject to copyright restrictions of the respective repositories: https://www.morphosource.org/Detail/ProjectDetail/Show/project_id/1004.

## Abbreviations

3D GM: Three-dimensional geometric morphometrics
CT: Computed tomography
CVA: Canonical variate analysis
*K*_mult_: Multivariate phylogenetic signal
lnCS: Natural log-transformed centroid size
PC: Principal component
Pcoords: Procrustes coordinates
PGLS: Phylogenetic generalised least squares
UPGMA: Unweighted pair group method with arithmetic mean
