# Supplementary material for: Functional ecological convergence between the thylacine and small prey-focused canids
Source: BMC Ecol Evol. 2021 Apr 21;21:58. doi: 10.1186/s12862-021-01788-8 (PMC8059158; doi:10.1186/s12862-021-01788-8)
Supplement: Supplementary file 1 — Additional file 1: Consists of all supplementary figures and tables cited in the manuscript, and supplementary references for the diet, body mass, prey size data, and phylogenetic tree (found at: https://figshare.com/projects/Convergence_between_the_thylacine_and_small_prey-focused_canids/84905).Figs S1–6. PC scores 1–4 mapped onto phylogeny for the total cranial, facial patch, and neurocranial patch datasets.Table S1. CVA discrimination coefficients. Table S2. Spearman correlation and Wilcoxon ranked sum results. TablesS3–5. Results of the C1–C4 and phenotypic angle θ convergence tests for the total cranial, facial patch, and neurocranial patch datasets. Table S6. List of specimens, and Morphosource DOI for each surface mesh file. Table S7. References for composite phylogenetic tree. Table S8. 3D GM landmark definitions and protocol. Table S9. References for body mass and diet. [file 12862_2021_1788_MOESM1_ESM.pdf]

Additional File 1: Supplemental material for:

Functional ecological convergence between the thylacine and small prey-focused canids.

Rovinsky, DS, Evans, AR, Adams JW.

Figure S1: Cranial PCs 1-2 mapped onto phylogeny 2

Figure S2: Cranial PCs 3-4 mapped onto phylogeny 3

Figure S3: Facial PCs 1-2 mapped onto phylogeny 4

Figure S4: Facial PCs 3-4 mapped onto phylogeny 5

Figure S5: Neurocranial PCs 1-2 mapped onto phylogeny 6

Figure S6: Neurocranial PCs 3-4 mapped onto phylogeny 7

Table S1: CVA coefficients and discrimination 8

Table S2: Spearman correlation and Wilcoxon ranked sum test results 9

Table S3: Convergence results - cranial 10

Table S4: Convergence results - facial 11

Table S5: Convergence results - neurocranial 12

Table S6: Specimen list 13

Table S7: Phylogenetic tree references 21

Table S8: Landmark and semilandmark list and protocol 22

Table S9: References for body mass and diet 28

Additional data (Morphologika files, phylogenetic tree, diet & body mass data, data for replication of analyses) and R code can be found at: Figshare Project 84905

https://figshare.com/projects/Convergence_between_the_thylacine_and_small_prey-focused_canids/84905

Surface meshes can be found at: Morphosource Project P1004.

https://www.morphosource.org/Detail/ProjectDetail/Show/project_id/1004

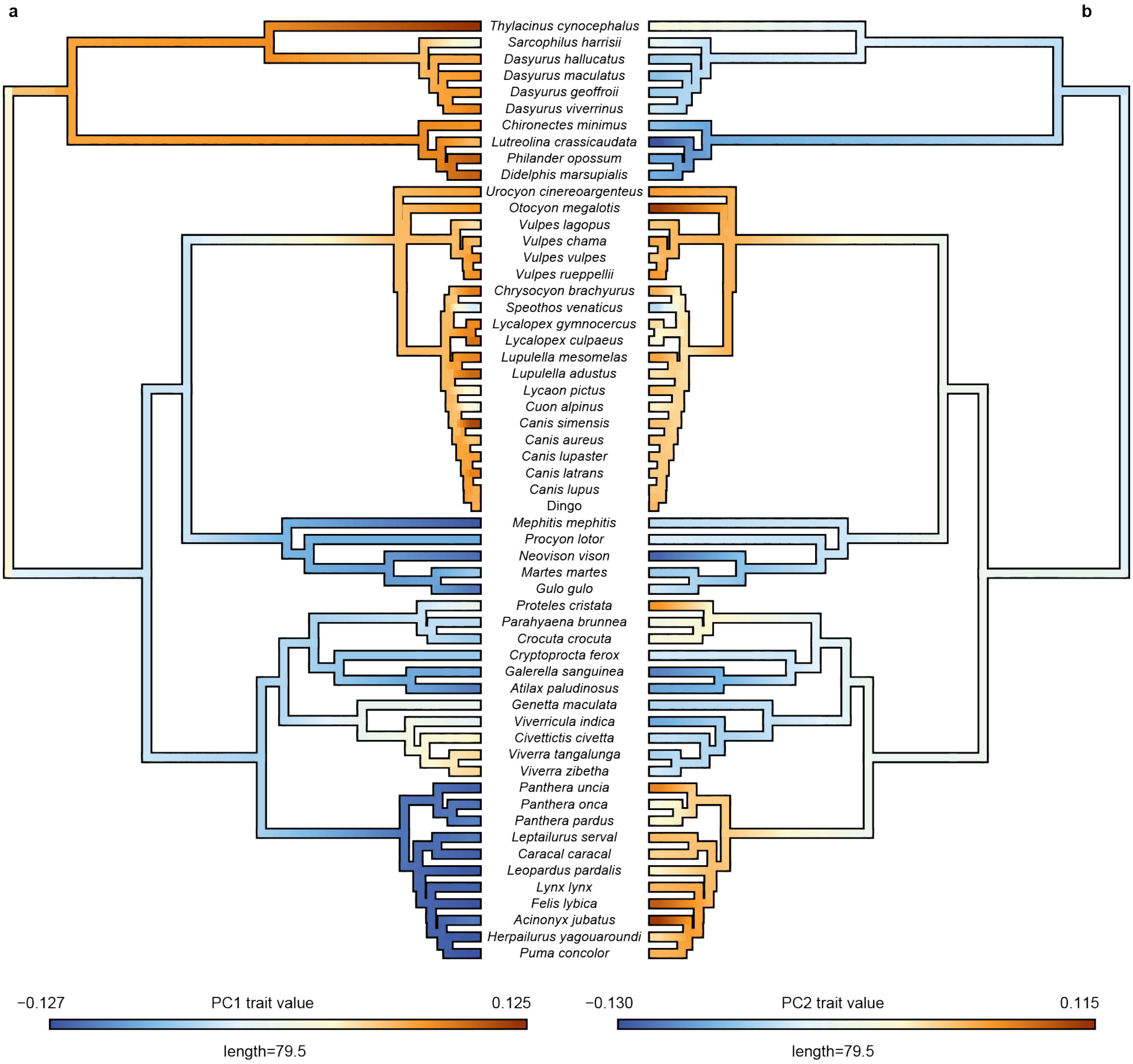

Figure S1. Total cranium dataset PC1 (a) and PC2 (b) mapped onto phylogeny.

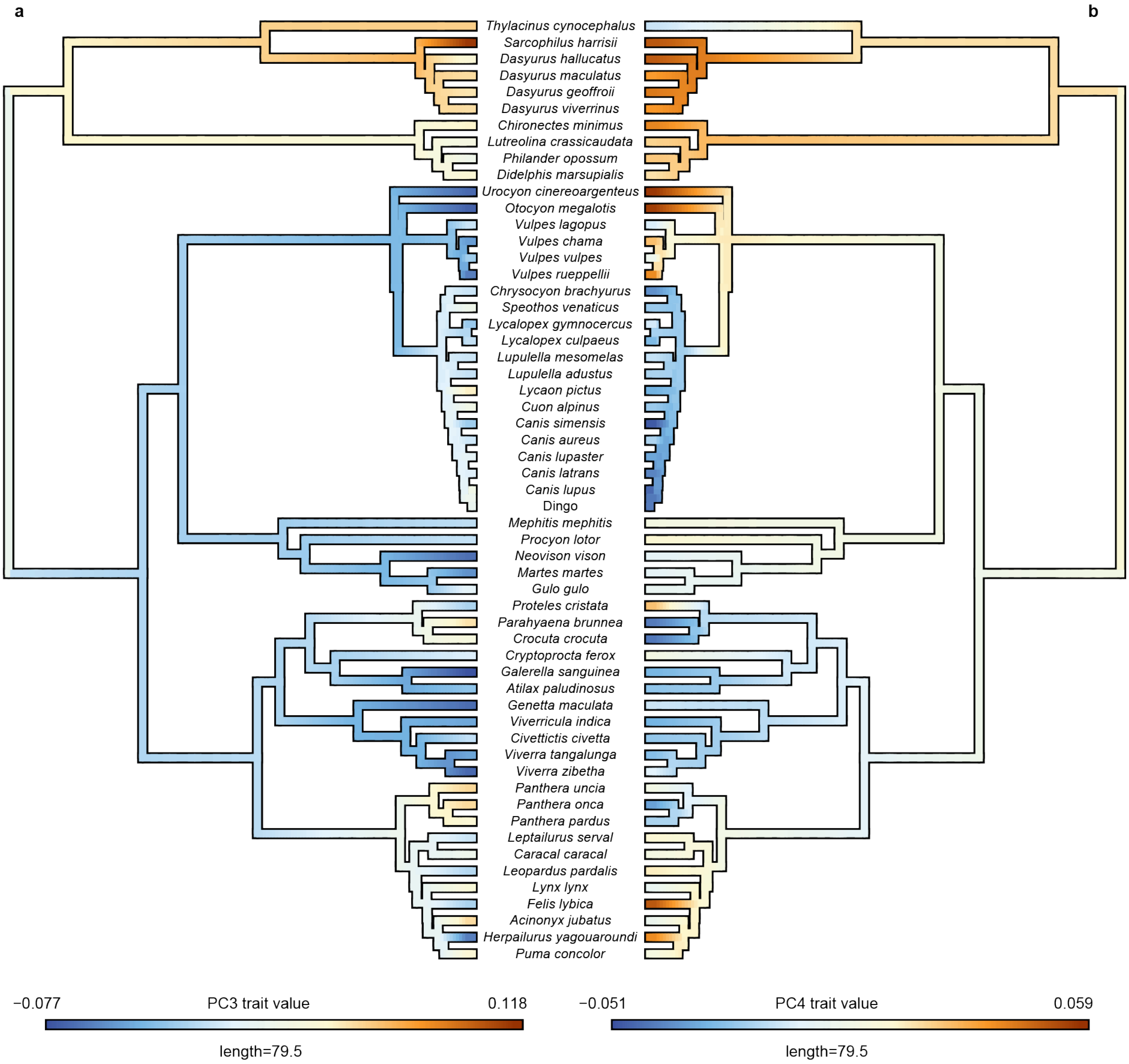

Figure S2. Total cranium dataset PC3 (a) and PC4 (b) mapped onto phylogeny.

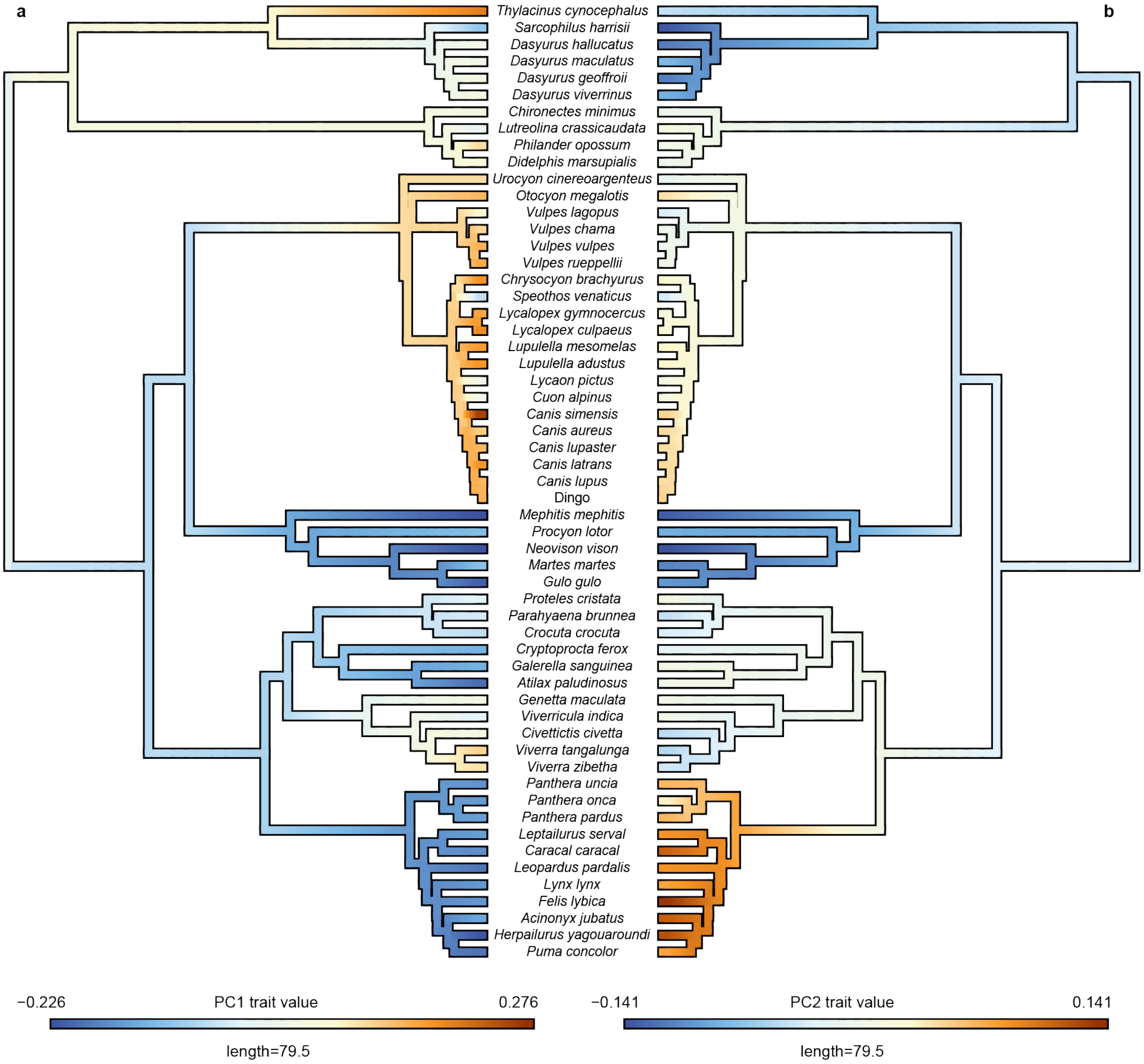

Figure S3. Facial patch data subset PC1 (a) and PC2 (b) mapped onto phylogeny.

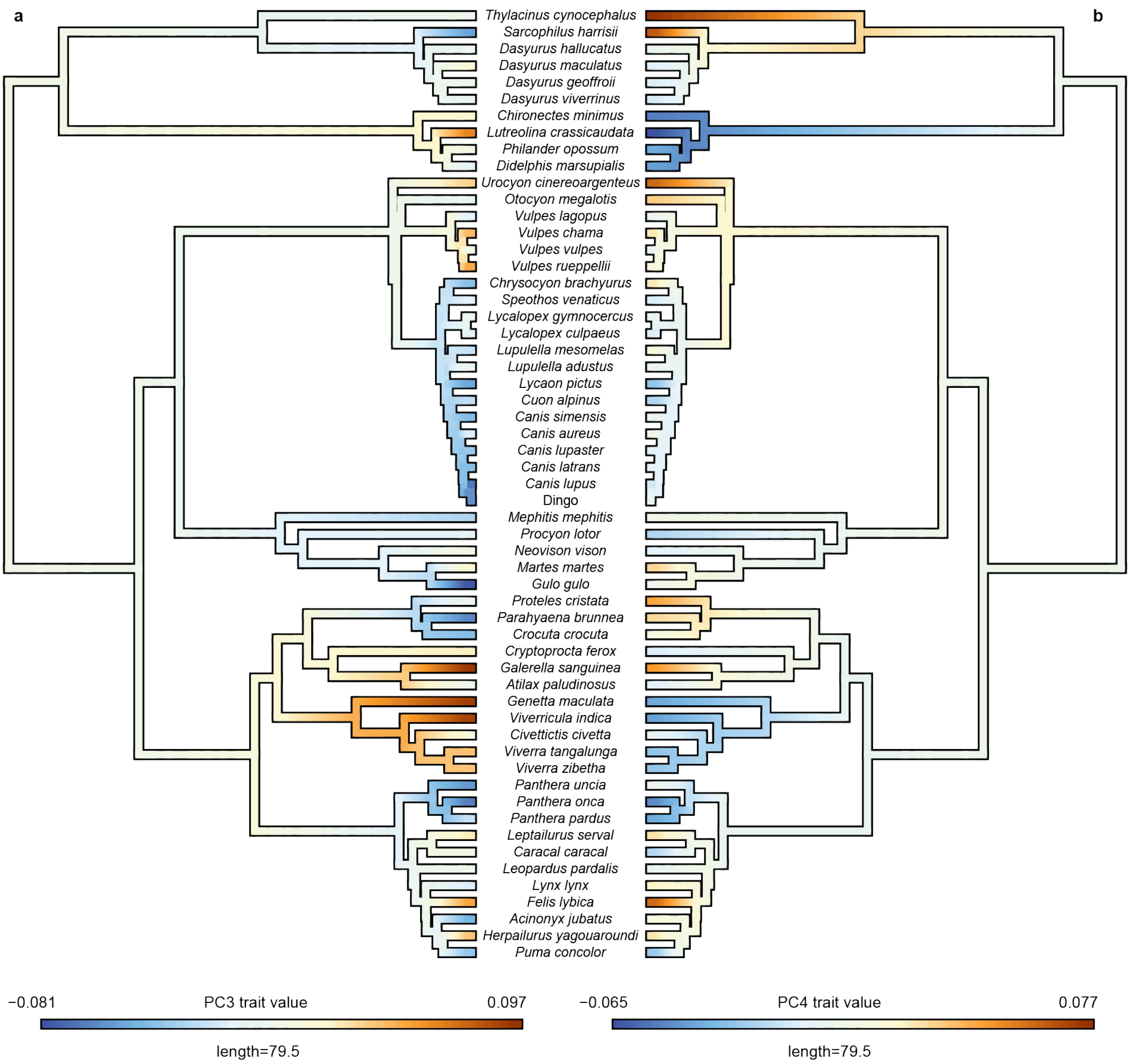

Figure S4. Facial patch data subset PC3 (a) and PC4 (b) mapped onto phylogeny.

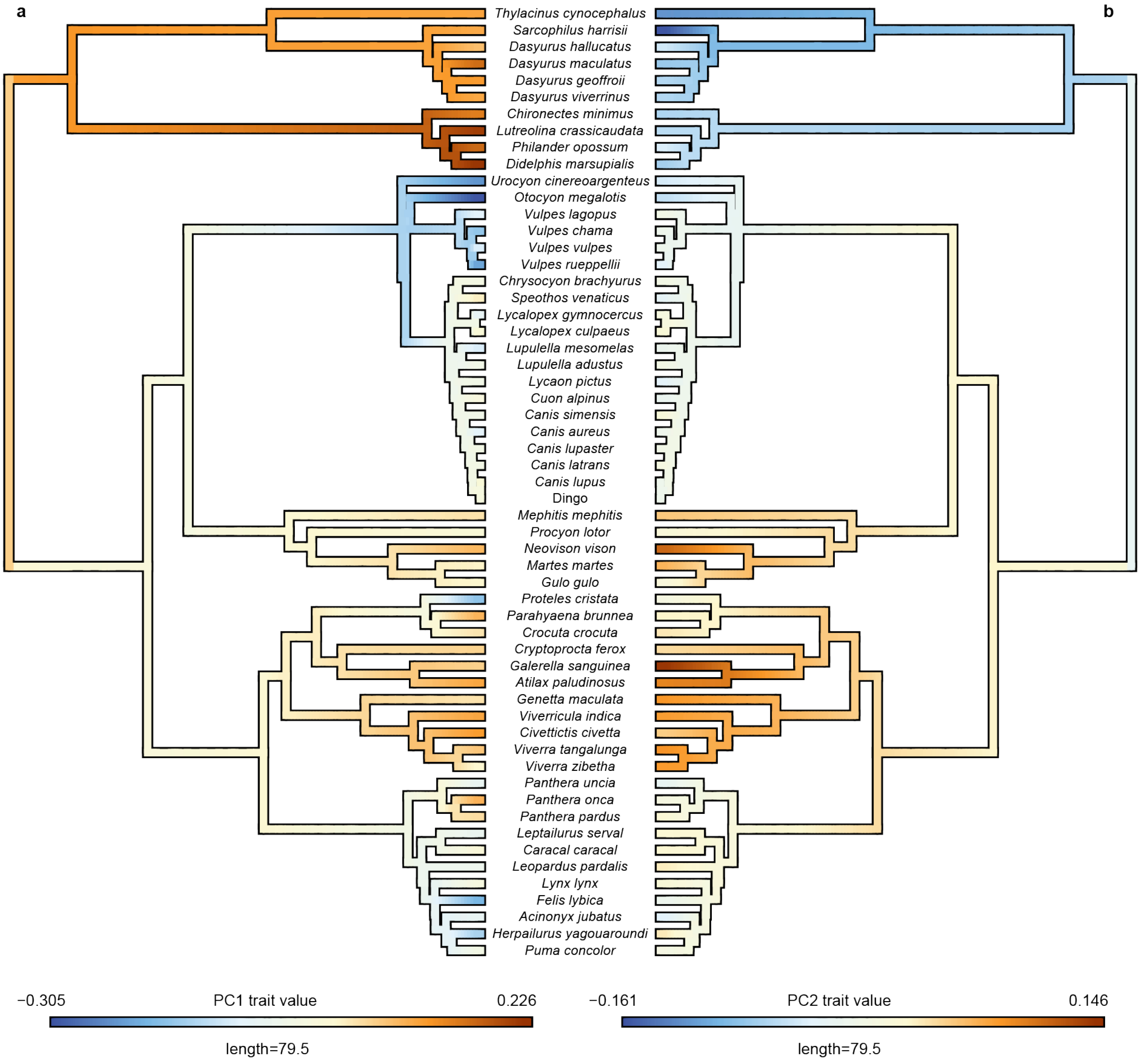

Figure S5. Neurocranial patch data subset PC1 (a) and PC2 (b) mapped onto phylogeny.

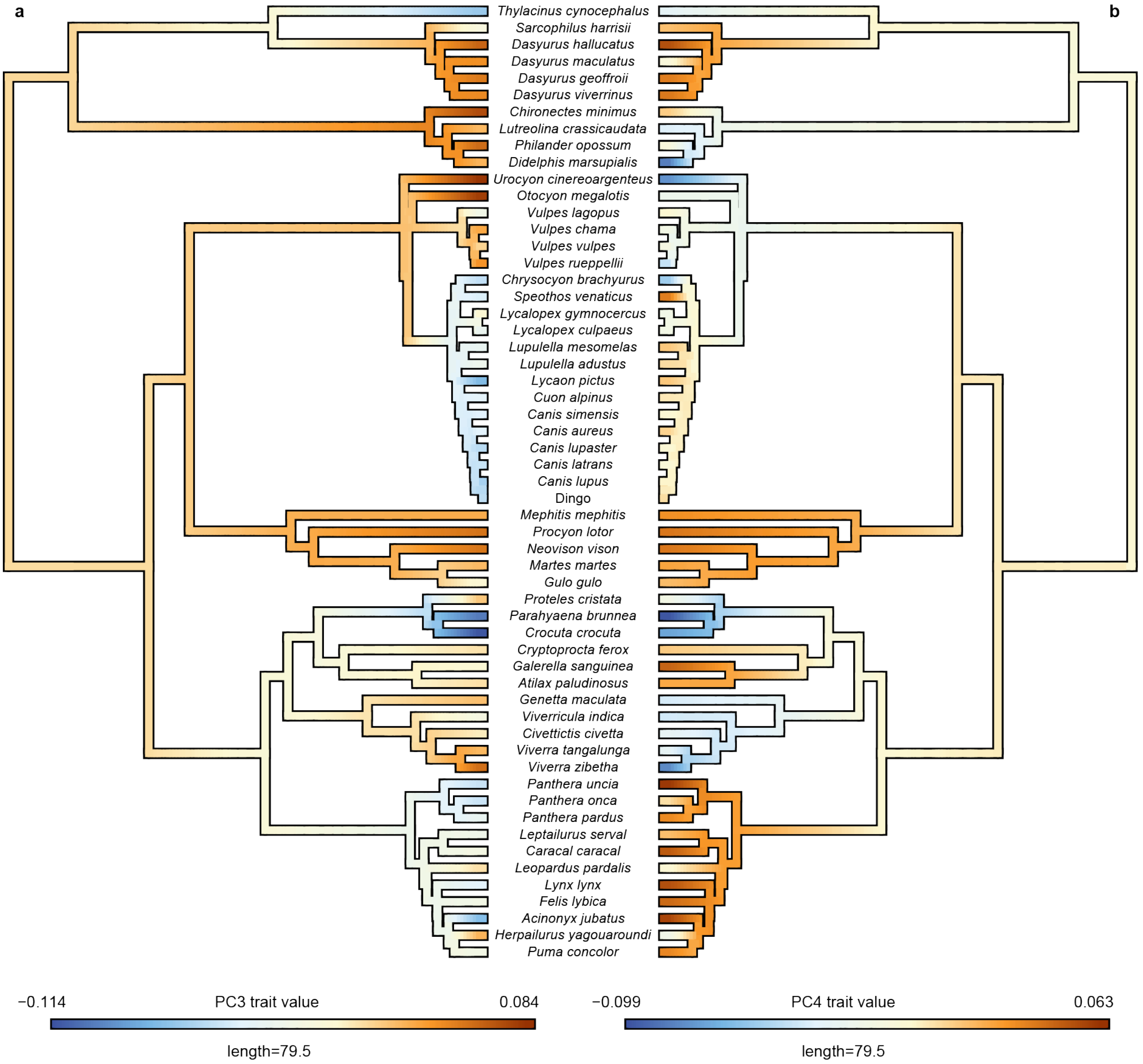

Figure S6. Neurocranial patch data subset PC3 (a) and PC4 (b) mapped onto phylogeny.

Table S1. Coefficients of linear discriminants.

PC > 1% var.	Cranial set	Facial set	Neurocranial set
PC1	4.7400106	3.333185	-3.055865
PC2	-3.9941711	3.735905	8.137784
PC3	18.2319980	20.247608	17.191593
PC4	16.3022083	7.825238	-12.262340
PC5	-0.8015981	12.914303	-3.553954
PC6	2.4844696	-5.584836	15.496704
PC7	14.7576553	-13.851487	17.271646
PC8	23.2724753	NA	16.859289
PC9	-26.2385031	NA	-14.304833
PC10	-26.7634082	NA	NA

Linear discriminants of all principal components >1% variance. The total cranial set shows 87.4% correct attribution, placing the thylacine into the small prey group (89.4% posterior probability). The facial set shows 80.1% correct attribution, placing the thylacine into the small prey group (95.9% posterior probability). The neurocranial set shows 87.4% correct attribution, placing the thylacine into the large prey group (87.2% posterior probability).

Table S2. Spearman's correlation and Wilcoxon rank sum results on PC1—4 of the total cranial dataset.

		Spearman's Correlation		Wilcoxon Rank Sum
	Cranium	*P*	rho	*P*
PC1	total	**0.001**	0.240	0.378
	placental	**0.004**	0.220	
	marsupial	**0.003**	0.488	
PC2	total	0.316	0.070	0.107
	placental	0.881	0.012	
	marsupial	0.194	0.222	
PC3	total	**<0.001**	0.460	**0.001**
	placental	**<0.001**	0.564	
	marsupial	**0.007**	0.441	
PC4	total	**<0.001**	0.279	0.210
	placental	**0.002**	0.240	
	marsupial	**0.001**	0.521	
	Face			
PC1	total	**0.003**	0.208	**<0.001**
	placental	**0.015**	0.187	
	marsupial	**0.023**	0.377	
PC2	total	**<0.001**	0.257	0.178
	placental	**<0.001**	0.282	
	marsupial	**0.001**	0.545	
PC3	total	**<0.001**	0.359	**<0.001**
	placental	**<0.001**	0.438	
	marsupial	0.068	0.308	
PC4	total	**<0.001**	0.248	**<0.001**
	placental	**0.022**	0.176	
	marsupial	**0.001**	0.524	
	Neurocranium			
PC1	total	0.184	0.093	0.378
	placental	**0.001**	0.256	
	marsupial	0.160	0.239	
PC2	total	0.573	0.040	0.107
	placental	0.175	0.105	
	marsupial	0.448	0.130	
PC3	total	**<0.001**	0.476	**0.001**
	placental	**<0.001**	0.500	
	marsupial	0.683	0.070	
PC4	total	**<0.001**	0.376	0.210
	placental	**<0.001**	0.454	
	marsupial	0.253	0.196	

Wilcoxon test is between prey size category (< 45% predator mass and > 45% predator mass) in total data set (test for PC differences between prey categories). Bolded values are significant after adjustment for multiple comparisons by the Benjamini-Hochberg method, α = 0.10.

Table S3. Total cranium C1–C4 and θ˚ convergence results.

	C1—4								θ˚	
**Species**	**C1**	***P***	**C2**	***P***	**C3**	***P***	**C4**	***P***	**θ**	***P***
Canis aureus	0.058	0.471	0.008	0.520	0.016	0.634	0.002	0.653	57.4˚	**0.008**
Canis latrans	0.452	**0.003**	0.093	**<0.001**	0.133	0.066	0.024	0.045	41.4˚	**0.001**
Canis lupaster	0.206	0.112	0.030	0.138	0.054	0.324	0.008	0.365	41.9˚	**0.003**
Canis lupus	0.201	0.139	0.030	0.159	0.051	0.368	0.008	0.392	43.7˚	**0.004**
Canis simensis	0.135	0.213	0.019	0.235	0.034	0.458	0.005	0.472	45.8˚	**0.009**
Chrysocyon brachyurus	0.507	**0.001**	0.116	**<0.001**	0.175	0.017	0.030	0.013	44.4˚	**0.006**
Cuon alpinus	0.327	**0.025**	0.072	**0.002**	0.110	0.107	0.019	0.093	76.1˚	**0.049**
Dingo	0.182	0.144	0.027	0.160	0.047	0.384	0.007	0.397	50.0˚	**0.009**
Lupulella adustus	0.537	**0.001**	0.122	**<0.001**	0.172	0.023	0.031	0.016	35.4˚	**0.001**
Lupulella mesomelas	0.524	**0.001**	0.134	**<0.001**	0.182	0.011	0.035	0.007	46.0˚	**0.006**
Lycalopex culpaeus	0.489	**0.003**	0.101	**0.001**	0.159	0.027	0.026	0.034	38.1˚	**0.003**
Lycalopex gymnocercus	0.417	**0.005**	0.089	**0.003**	0.126	0.068	0.023	0.046	51.7˚	**0.005**
Lycaon pictus	0.194	0.104	0.036	0.080	0.057	0.298	0.009	0.282	71.3˚	**0.027**
Otocyon megalotis	0.078	0.394	0.018	0.309	0.029	0.496	0.005	0.486	81.1˚	**0.053**
Proteles cristata	0.195	0.121	0.046	0.050	0.081	0.202	0.012	0.221	96.1˚	0.150
Urocyon cinereoargenteus	0.046	0.439	0.009	0.430	0.015	0.547	0.002	0.538	76.5˚	**0.045**
Vulpes chama	0.159	0.181	0.030	0.131	0.048	0.379	0.008	0.362	74.5˚	**0.043**
Vulpes lagopus	0.169	0.149	0.028	0.133	0.052	0.317	0.007	0.356	63.6˚	**0.022**
Vulpes rueppellii	0.090	0.350	0.017	0.293	0.026	0.514	0.004	0.500	72.3˚	**0.036**
Vulpes vulpes	0.333	**0.023**	0.059	**0.012**	0.094	0.140	0.015	0.135	49.3˚	**0.006**

C1 values are the scaled phenotypic distances closed between the species and the thylacine, θ are the search.conv angles between the multivariate phenotypic vectors of the same. Significant *P*-values in bold; significance adjusted for multiple comparisons by the Benjamini-Hochberg method, α = 0.10.

Table S4. Facial patch subset C1–C4 and θ˚ convergence results.

	C1—4								θ˚	
**Species**	**C1**	***P***	**C2**	***P***	**C3**	***P***	**C4**	***P***	**θ**	***P***
Canis aureus	0.194	0.251	0.044	0.230	0.073	0.413	0.009	0.478	49.6˚	**0.026**
Canis latrans	0.305	0.127	0.071	0.090	0.092	0.318	0.015	0.319	42.6˚	**0.020**
Canis lupaster	0.315	0.133	0.083	0.072	0.132	0.202	0.017	0.273	49.3˚	**0.022**
Canis lupus	0.236	0.220	0.058	0.162	0.088	0.344	0.012	0.399	51.3˚	**0.032**
Canis simensis	0.237	0.200	0.054	0.164	0.078	0.377	0.011	0.406	37.7˚	**0.007**
Chrysocyon brachyurus	0.566	**0.013**	0.185	**0.001**	0.235	0.031	0.038	0.043	36.2˚	**0.004**
Cuon alpinus	0.176	0.212	0.060	0.045	0.102	0.206	0.011	0.317	76.6˚	0.104
Dingo	0.305	0.114	0.077	0.070	0.114	0.230	0.016	0.279	47.2˚	**0.020**
Lupulella adustus	0.642	**0.007**	0.260	**<0.001**	0.279	0.016	0.054	**0.010**	37.6˚	**0.009**
Lupulella mesomelas	0.627	**0.005**	0.256	**<0.001**	0.273	0.013	0.053	**0.007**	39.6˚	**0.015**
Lycalopex culpaeus	0.378	0.076	0.090	0.050	0.144	0.168	0.019	0.241	38.4˚	**0.008**
Lycalopex gymnocercus	0.565	**0.007**	0.190	**0.001**	0.212	0.050	0.039	0.029	37.1˚	**0.012**
Lycaon pictus	0.100	0.446	0.028	0.372	0.035	0.574	0.006	0.563	90.1˚	0.155
Otocyon megalotis	0.506	0.013	0.180	**<0.001**	0.232	0.028	0.037	0.025	48.9˚	**0.022**
Proteles cristata	0.189	0.226	0.057	0.120	0.097	0.278	0.011	0.363	81.9˚	**0.090**
Urocyon cinereoargenteus	0.468	**0.024**	0.138	**0.009**	0.193	0.080	0.028	0.101	38.9˚	**0.012**
Vulpes chama	0.381	0.055	0.106	**0.024**	0.135	0.178	0.022	0.157	46.6˚	**0.027**
Vulpes lagopus	0.133	0.311	0.028	0.316	0.043	0.476	0.006	0.500	40.9˚	**0.018**
Vulpes rueppellii	0.337	0.090	0.076	0.068	0.102	0.289	0.016	0.284	40.9˚	**0.017**
Vulpes vulpes	0.356	0.076	0.076	0.070	0.127	0.213	0.016	0.304	33.5˚	**0.004**

C1 values are the scaled phenotypic distances closed between the species and the thylacine, θ are the search.conv angles between the multivariate phenotypic vectors of the same. Significant *P*-values in bold; significance adjusted for multiple comparisons by the Benjamini-Hochberg method, $\alpha = 0.10$.

Table S5. Neurocranium patch subset C1–C4 and θ˚ convergence results.

	C1—4								θ˚	
**Species**	**C1**	***P***	**C2**	***P***	**C3**	***P***	**C4**	***P***	**θ**	***P***
Canis aureus	0.070	0.451	0.019	0.362	0.024	0.598	0.004	0.604	110.4˚	0.179
Canis latrans	0.384	0.015	0.142	**<0.001**	0.137	0.087	0.027	0.052	107.8˚	0.171
Canis lupaster	0.230	0.102	0.064	**0.039**	0.078	0.241	0.012	0.252	94.5˚	0.122
Canis lupus	0.318	0.043	0.088	**0.012**	0.097	0.182	0.017	0.164	72.1˚	0.051
Canis simensis	0.145	0.243	0.040	0.151	0.051	0.401	0.008	0.416	119.5˚	0.201
Chrysocyon brachyurus	0.271	0.062	0.075	**0.015**	0.090	0.190	0.014	0.193	80.7˚	0.073
Cuon alpinus	0.258	0.081	0.071	**0.028**	0.087	0.227	0.013	0.239	87.5˚	0.098
Dingo	0.271	0.082	0.075	**0.024**	0.088	0.220	0.014	0.231	84.8˚	0.091
Lupulella adustus	0.196	0.143	0.054	0.060	0.061	0.319	0.010	0.287	116.4˚	0.201
Lupulella mesomelas	0.076	0.420	0.022	0.295	0.023	0.600	0.004	0.569	114.6˚	0.192
Lycalopex culpaeus	0.515	**0.004**	0.226	**<0.001**	0.215	0.009	0.043	**0.002**	96.4˚	0.141
Lycalopex gymnocercus	0.014	0.694	0.004	0.660	0.004	0.737	0.001	0.723	128.1˚	0.282
Lycaon pictus	0.243	0.091	0.067	**0.029**	0.079	0.226	0.013	0.213	82.7˚	0.070
Otocyon megalotis	0.000	0.999	0.000	0.999	0.000	0.999	0.000	0.999	117.6˚	0.199
Proteles cristata	0.000	0.999	0.000	0.999	0.000	0.999	0.000	0.999	121.2˚	0.227
Urocyon cinereoargenteus	0.000	0.999	0.000	0.999	0.000	0.999	0.000	0.999	114.3˚	0.197
Vulpes chama	0.000	0.999	0.000	0.999	0.000	0.999	0.000	0.999	121.3˚	0.223
Vulpes lagopus	0.174	0.180	0.055	0.068	0.074	0.293	0.010	0.298	117.3˚	0.216
Vulpes rueppellii	0.000	0.999	0.000	0.999	0.000	0.999	0.000	0.999	118.0˚	0.215
Vulpes vulpes	0.189	0.166	0.062	**0.041**	0.062	0.312	0.012	0.257	125.5˚	0.237

C1 values are the scaled phenotypic distances closed between the species and the thylacine, θ are the search.conv angles between the multivariate phenotypic vectors of the same. Significant *P*-values in bold; significance adjusted for multiple comparisons by the Benjamini-Hochberg method, $\alpha = 0.10$.

Table S6. Specimen list and Morphosource DOIs. Institution abbreviations: AM (Australian Museum, Sydney, Australia), AMNH (American Museum of Natural History, New York, USA), DMNH (Ditsong National Museum of Natural History, Pretoria, South Africa), MSU (Michigan State University, Lansing, USA), NHMUK (Natural History Museum, London, UK), NMV (National Museums Victoria, Melbourne, Australia), SAM (South Australian Museum, Adelaide, Australia), SMNS (State Museum of Natural History, Stuttgart, Germany), TMAG (Tasmanian Museum and Art Gallery, Hobart, Australia), UMZC (University Museum of Zoology, Cambridge, UK), USNM (Smithsonian National Museum of Natural History, Washington, D.C., USA), WAM (Western Australian Museum, Perth, Australia), ZMB (Natural History Museum, Berlin, Germany).

Specimen	Sex	Species	3D Mesh Source	DOI
DMNH 1217	F	*Acinonyx jubatus*	CT	https://doi.org/10.17602/M2/M114684
DMNH 1269	U	*Acinonyx jubatus*	CT	https://doi.org/10.17602/M2/M114685
DMNH 162	M	*Acinonyx jubatus*	CT	https://doi.org/10.17602/M2/M117243
DMNH 25602	M	*Acinonyx jubatus*	CT	https://doi.org/10.17602/M2/M114686
DMNH 20426	M	*Atilax paludinosus*	CT	https://doi.org/10.17602/M2/M114721
DMNH 5330	M	*Atilax paludinosus*	CT	https://doi.org/10.17602/M2/M114722
DMNH 6191	F	*Atilax paludinosus*	CT	https://doi.org/10.17602/M2/M114723
DMNH 9019	F	*Atilax paludinosus*	CT	https://doi.org/10.17602/M2/M114724
AM M 42917	U	*Canis aureus*	Artec Spider	https://doi.org/10.17602/M2/M114633
NHMUK ZD 1869.8.13.3	U	*Canis aureus*	Artec Spider	https://doi.org/10.17602/M2/M114671
SMNS Z-MAM 5014	F	*Canis aureus*	Artec Spider	https://doi.org/10.17602/M2/M115290
SMNS Z-MAM 5016	M	*Canis aureus*	Artec Spider	https://doi.org/10.17602/M2/M115292
NHMUK ZD 1987.1	F	*Canis latrans*	Artec Spider	https://doi.org/10.17602/M2/M114672
SMNS Z-MAM 18994	U	*Canis latrans*	Artec Spider	https://doi.org/10.17602/M2/M115316
SMNS Z-MAM 51589	U	*Canis latrans*	Artec Spider	https://doi.org/10.17602/M2/M115317
ZMB Mam 35327	F	*Canis latrans*	Artec Spider	https://doi.org/10.17602/M2/M115893
SMNS Z-MAM 1174	F	*Canis lupaster*	Artec Spider	https://doi.org/10.17602/M2/M115283
SMNS Z-MAM 19006	M	*Canis lupaster*	Artec Spider	https://doi.org/10.17602/M2/M115284
SMNS Z-MAM 31972	M	*Canis lupaster*	Artec Spider	https://doi.org/10.17602/M2/M115288
SMNS Z-MAM 51593	F	*Canis lupaster*	Artec Spider	https://doi.org/10.17602/M2/M115289
SMNS Z-MAM 004973	F	*Canis lupus*	Artec Spider	https://doi.org/10.17602/M2/M115318
USNM 168440	M	*Canis lupus*	Artec Spider	https://doi.org/10.17602/M2/M117236

ZMB Mam 22392	U	*Canis lupus*	Artec Spider	https://doi.org/10.17602/M2/M115894
ZMB Mam 28797	F	*Canis lupus*	Artec Spider	https://doi.org/10.17602/M2/M115946
NHMUK ZD 1924.8.7.10	F	*Canis simensis*	Artec Spider	https://doi.org/10.17602/M2/M114673
NHMUK ZD 1936.5.20.4	F	*Canis simensis*	Artec Spider	https://doi.org/10.17602/M2/M114674
ZMB Mam 52489	M	*Canis simensis*	Artec Spider	https://doi.org/10.17602/M2/M115947
ZMB Mam 69300	U	*Canis simensis*	Artec Spider	https://doi.org/10.17602/M2/M115948
DMNH 15719	M	*Caracal caracal*	CT	https://doi.org/10.17602/M2/M114727
DMNH 873	M	*Caracal caracal*	CT	https://doi.org/10.17602/M2/M117244
DMNH 876	F	*Caracal caracal*	CT	https://doi.org/10.17602/M2/M114728
DMNH 877	F	*Caracal caracal*	CT	https://doi.org/10.17602/M2/M114729
AMNH 37482	M	*Chironectes minimus*	Artec Spider	https://doi.org/10.17602/M2/M114637
AMNH 96760	M	*Chironectes minimus*	Artec Spider	https://doi.org/10.17602/M2/M114638
USNM 305167	F	*Chironectes minimus*	Artec Spider	https://doi.org/10.17602/M2/M117237
USNM 517239	F	*Chironectes minimus*	Artec Spider	https://doi.org/10.17602/M2/M117238
SMNS Z-MAM 50753	F	*Chrysocyon brachyurus*	Artec Spider	https://doi.org/10.17602/M2/M115319
ZMB Mam 44198	M	*Chrysocyon brachyurus*	Artec Spider	https://doi.org/10.17602/M2/M115949
ZMB Mam 44989	U	*Chrysocyon brachyurus*	Artec Spider	https://doi.org/10.17602/M2/M115950
ZMB Mam 62102	F	*Chrysocyon brachyurus*	Artec Spider	https://doi.org/10.17602/M2/M115951
DMNH 1190	M	*Civettictis civetta*	CT	https://doi.org/10.17602/M2/M114733
DMNH 17711	F	*Civettictis civetta*	CT	https://doi.org/10.17602/M2/M114734
DMNH 26705	F	*Civettictis civetta*	CT	https://doi.org/10.17602/M2/M114735
DMNH 3174	M	*Civettictis civetta*	CT	https://doi.org/10.17602/M2/M114736
DMNH 19370	M	*Crocuta crocuta*	CT	https://doi.org/10.17602/M2/M114737
DMNH 3269	F	*Crocuta crocuta*	CT	https://doi.org/10.17602/M2/M114738
SMNS Z-MAM 31174	U	*Crocuta crocuta*	Artec Spider	https://doi.org/10.17602/M2/M115320
SMNS Z-MAM 8060	M	*Crocuta crocuta*	Artec Spider	https://doi.org/10.17602/M2/M115321
AMNH 100463	U	*Cryptoprocta ferox*	Artec Spider	https://doi.org/10.17602/M2/M114639
AMNH 188213	M	*Cryptoprocta ferox*	Artec Spider	https://doi.org/10.17602/M2/M114640
AMNH 199544	U	*Cryptoprocta ferox*	Artec Spider	https://doi.org/10.17602/M2/M114641
SMNS Z-MAM 21615	U	*Cryptoprocta ferox*	Artec Spider	https://doi.org/10.17602/M2/M115322

ZMB Mam 13524	M	*Cuon alpinus*	Artec Spider	https://doi.org/10.17602/M2/M115952
ZMB Mam 52519	F	*Cuon alpinus*	Artec Spider	https://doi.org/10.17602/M2/M115953
ZMB Mam 56621	M	*Cuon alpinus*	Artec Spider	https://doi.org/10.17602/M2/M115954
ZMB Mam 56622	F	*Cuon alpinus*	Artec Spider	https://doi.org/10.17602/M2/M115955
NMV C 31560	M	*Dasyurus geoffroii*	Artec Spider	https://doi.org/10.17602/M2/M115136
SAM M 79	F	*Dasyurus geoffroii*	Artec Spider	https://doi.org/10.17602/M2/M115272
WAM M 1106	M	*Dasyurus geoffroii*	Artec Spider	https://doi.org/10.17602/M2/M115881
WAM M 1294	F	*Dasyurus geoffroii*	Artec Spider	https://doi.org/10.17602/M2/M115882
NMV C 17713	M	*Dasyurus hallucatus*	Artec Spider	https://doi.org/10.17602/M2/M115137
WAM M 12410	F	*Dasyurus hallucatus*	Artec Spider	https://doi.org/10.17602/M2/M115883
WAM M 7161	M	*Dasyurus hallucatus*	Artec Spider	https://doi.org/10.17602/M2/M115884
WAM M 7168	F	*Dasyurus hallucatus*	Artec Spider	https://doi.org/10.17602/M2/M115885
NMV C 14721	M	*Dasyurus maculatus*	Artec Spider	https://doi.org/10.17602/M2/M115138
NMV C 6107	F	*Dasyurus maculatus*	Artec Spider	https://doi.org/10.17602/M2/M115139
NMV C 6119	F	*Dasyurus maculatus*	Artec Spider	https://doi.org/10.17602/M2/M115140
WAM M 3470	M	*Dasyurus maculatus*	Artec Spider	https://doi.org/10.17602/M2/M115886
NMV DTC 530	M	*Dasyurus viverrinus*	Artec Spider	https://doi.org/10.17602/M2/M115142
SAM M 7217	F	*Dasyurus viverrinus*	Artec Spider	https://doi.org/10.17602/M2/M115275
SAM M 7221	M	*Dasyurus viverrinus*	Artec Spider	https://doi.org/10.17602/M2/M115276
WAM M 6559	F	*Dasyurus viverrinus*	Artec Spider	https://doi.org/10.17602/M2/M115887
AMNH 133002	M	*Didelphis marsupialis*	Artec Spider	https://doi.org/10.17602/M2/M114642
AMNH 133007	F	*Didelphis marsupialis*	Artec Spider	https://doi.org/10.17602/M2/M114643
NHMUK ZD 1984.374	F	*Didelphis marsupialis*	Artec Spider	https://doi.org/10.17602/M2/M114675
NHMUK ZD 1899.8.1.18	M	*Didelphis marsupialis*	Artec Spider	https://doi.org/10.17602/M2/M114676
AM M 18851	M	Dingo	Artec Spider	https://doi.org/10.17602/M2/M114634
AM M 18970	F	Dingo	Artec Spider	https://doi.org/10.17602/M2/M114635
WAM M 3831	F	Dingo	Artec Spider	https://doi.org/10.17602/M2/M115879
WAM M 5261	M	Dingo	Artec Spider	https://doi.org/10.17602/M2/M115880
DMNH 10927	M	*Felis lybica*	CT	https://doi.org/10.17602/M2/M114739
DMNH 10930	F	*Felis lybica*	CT	https://doi.org/10.17602/M2/M114740

DMNH 17016	M	*Felis lybica*	CT	https://doi.org/10.17602/M2/M114797
DMNH 6154	F	*Felis lybica*	CT	https://doi.org/10.17602/M2/M114798
SMNS Z-MAM 24104	F	*Galerella sanguinea*	Artec Spider	https://doi.org/10.17602/M2/M115323
SMNS Z-MAM 4396	F	*Galerella sanguinea*	Artec Spider	https://doi.org/10.17602/M2/M115324
SMNS Z-MAM 4398	M	*Galerella sanguinea*	Artec Spider	https://doi.org/10.17602/M2/M115325
SMNS Z-MAM 4399	M	*Galerella sanguinea*	Artec Spider	https://doi.org/10.17602/M2/M115326
SMNS Z-MAM 4412	F	*Genetta maculata*	Artec Spider	https://doi.org/10.17602/M2/M115442
SMNS Z-MAM 4416	F	*Genetta maculata*	Artec Spider	https://doi.org/10.17602/M2/M115443
SMNS Z-MAM 4425	M	*Genetta maculata*	Artec Spider	https://doi.org/10.17602/M2/M115444
SMNS Z-MAM 4427	M	*Genetta maculata*	Artec Spider	https://doi.org/10.17602/M2/M115445
MSU 10149	F	*Gulo gulo*	Artec Spider	https://doi.org/10.17602/M2/M114831
MSU 3844	U	*Gulo gulo*	Artec Spider	https://doi.org/10.17602/M2/M114832
SMNS Z-MAM 51682	U	*Gulo gulo*	Artec Spider	https://doi.org/10.17602/M2/M115446
SMNS Z-MAM 6833	U	*Gulo gulo*	Artec Spider	https://doi.org/10.17602/M2/M115447
ZMB Mam 21295	M	*Herpailurus yagouaroundi*	Artec Spider	https://doi.org/10.17602/M2/M115956
ZMB Mam 21297	U	*Herpailurus yagouaroundi*	Artec Spider	https://doi.org/10.17602/M2/M115957
ZMB Mam 5815	M	*Herpailurus yagouaroundi*	Artec Spider	https://doi.org/10.17602/M2/M115958
ZMB Mam 5816	F	*Herpailurus yagouaroundi*	Artec Spider	https://doi.org/10.17602/M2/M115959
SMNS Z-MAM 26251	U	*Leopardus pardalis*	Artec Spider	https://doi.org/10.17602/M2/M115448
SMNS Z-MAM 40178	U	*Leopardus pardalis*	Artec Spider	https://doi.org/10.17602/M2/M115449
SMNS Z-MAM 6970	F	*Leopardus pardalis*	Artec Spider	https://doi.org/10.17602/M2/M115450
DMNH 2295	M	*Leptailurus serval*	CT	https://doi.org/10.17602/M2/M114812
DMNH 93	U	*Leptailurus serval*	CT	https://doi.org/10.17602/M2/M114813
SMNS Z-MAM 18897	M	*Leptailurus serval*	Artec Spider	https://doi.org/10.17602/M2/M115451
SMNS Z-MAM 18903	F	*Leptailurus serval*	Artec Spider	https://doi.org/10.17602/M2/M115452
DMNH 1001	M	*Lupulella adustus*	CT	https://doi.org/10.17602/M2/M114725
DMNH 10907	M	*Lupulella adustus*	CT	https://doi.org/10.17602/M2/M114726
SMNS Z-MAM 19004	F	*Lupulella adustus*	Artec Spider	https://doi.org/10.17602/M2/M115281
SMNS Z-MAM 19005	F	*Lupulella adustus*	Artec Spider	https://doi.org/10.17602/M2/M115282
DMNH 12002	M	*Lupulella mesomelas*	CT	https://doi.org/10.17602/M2/M114730

DMNH 12013	F	*Lupulella mesomelas*	CT	https://doi.org/10.17602/M2/M114731
DMNH 24017	F	*Lupulella mesomelas*	CT	https://doi.org/10.17602/M2/M117245
DMNH 4424	M	*Lupulella mesomelas*	CT	https://doi.org/10.17602/M2/M114732
AMNH 210420	M	*Lutreolina crassicaudata*	Artec Spider	https://doi.org/10.17602/M2/M114644
AMNH 254513	F	*Lutreolina crassicaudata*	Artec Spider	https://doi.org/10.17602/M2/M114645
NHMUK ZD 1917.1.25.69	F	*Lutreolina crassicaudata*	Artec Spider	https://doi.org/10.17602/M2/M114677
NHMUK ZD 1920.12.18.36	M	*Lutreolina crassicaudata*	Artec Spider	https://doi.org/10.17602/M2/M114678
SMNS Z-MAM 5692	U	*Lycalopex culpaeus*	Artec Spider	https://doi.org/10.17602/M2/M115453
ZMB Mam 77063	F	*Lycalopex culpaeus*	Artec Spider	https://doi.org/10.17602/M2/M115960
ZMB Mam 77106	M	*Lycalopex culpaeus*	Artec Spider	https://doi.org/10.17602/M2/M115961
ZMB Mam 77108	F	*Lycalopex culpaeus*	Artec Spider	https://doi.org/10.17602/M2/M115962
ZMB Mam 77031	M	*Lycalopex gymnocercus*	Artec Spider	https://doi.org/10.17602/M2/M115963
ZMB Mam 77033	M	*Lycalopex gymnocercus*	Artec Spider	https://doi.org/10.17602/M2/M115964
ZMB Mam 77035	F	*Lycalopex gymnocercus*	Artec Spider	https://doi.org/10.17602/M2/M115965
ZMB Mam 77050	F	*Lycalopex gymnocercus*	Artec Spider	https://doi.org/10.17602/M2/M115966
DMNH 11895	U	*Lycaon pictus*	CT	https://doi.org/10.17602/M2/M114814
DMNH 2244	M	*Lycaon pictus*	CT	https://doi.org/10.17602/M2/M114815
DMNH 47678	M	*Lycaon pictus*	CT	https://doi.org/10.17602/M2/M114816
SMNS Z-MAM 4460	F	*Lycaon pictus*	Artec Spider	https://doi.org/10.17602/M2/M115456
SMNS Z-MAM 1870	F	*Lynx lynx*	Artec Spider	https://doi.org/10.17602/M2/M115454
SMNS Z-MAM 18860	F	*Lynx lynx*	Artec Spider	https://doi.org/10.17602/M2/M115455
ZMB Mam 16997	U	*Lynx lynx*	Artec Spider	https://doi.org/10.17602/M2/M115967
SMNS Z-MAM 21806	U	*Martes martes*	Artec Spider	https://doi.org/10.17602/M2/M115457
SMNS Z-MAM 21808	M	*Martes martes*	Artec Spider	https://doi.org/10.17602/M2/M115458
SMNS Z-MAM 46570	F	*Martes martes*	Artec Spider	https://doi.org/10.17602/M2/M115799
SMNS Z-MAM 47060	M	*Martes martes*	Artec Spider	https://doi.org/10.17602/M2/M115800
SMNS Z-MAM 21879	U	*Mephitis mephitis*	Artec Spider	https://doi.org/10.17602/M2/M115801
SMNS Z-MAM 21881	U	*Mephitis mephitis*	Artec Spider	https://doi.org/10.17602/M2/M115802
WAM M 245	M	*Mephitis mephitis*	Artec Spider	https://doi.org/10.17602/M2/M115888
SMNS Z-MAM 21831	U	*Neovison vison*	Artec Spider	https://doi.org/10.17602/M2/M115803

SMNS Z-MAM 32536	U	*Neovison vison*	Artec Spider	https://doi.org/10.17602/M2/M115804
SMNS Z-MAM 51570	U	*Neovison vison*	Artec Spider	https://doi.org/10.17602/M2/M115805
DMNH 16786	M	*Otocyon megalotis*	CT	https://doi.org/10.17602/M2/M114817
DMNH 27297	F	*Otocyon megalotis*	CT	https://doi.org/10.17602/M2/M114819
ZMB Mam 35581	U	*Panthera onca*	Artec Spider	https://doi.org/10.17602/M2/M115968
ZMB Mam 47996	M	*Panthera onca*	Artec Spider	https://doi.org/10.17602/M2/M115969
ZMB Mam 56263	U	*Panthera onca*	Artec Spider	https://doi.org/10.17602/M2/M115970
ZMB Mam 56264	U	*Panthera onca*	Artec Spider	https://doi.org/10.17602/M2/M115971
DMNH 13276	F	*Panthera pardus*	CT	https://doi.org/10.17602/M2/M114825
SMNS Z-MAM 18957	M	*Panthera pardus*	Artec Spider	https://doi.org/10.17602/M2/M115806
SMNS Z-MAM 18959	F	*Panthera pardus*	Artec Spider	https://doi.org/10.17602/M2/M115807
SMNS Z-MAM 6854	M	*Panthera pardus*	Artec Spider	https://doi.org/10.17602/M2/M115808
ZMB Mam 14501	F	*Panthera uncia*	Artec Spider	https://doi.org/10.17602/M2/M115972
ZMB Mam 18434	F	*Panthera uncia*	Artec Spider	https://doi.org/10.17602/M2/M115973
ZMB Mam 43160	M	*Panthera uncia*	Artec Spider	https://doi.org/10.17602/M2/M115974
ZMB Mam 47690	M	*Panthera uncia*	Artec Spider	https://doi.org/10.17602/M2/M115975
DMNH 2387	F	*Parahyaena brunnea*	CT	https://doi.org/10.17602/M2/M114823
DMNH 25430	M	*Parahyaena brunnea*	CT	https://doi.org/10.17602/M2/M114824
DMNH 56B	F	*Parahyaena brunnea*	CT	https://doi.org/10.17602/M2/M117239
DMNH 603	M	*Parahyaena brunnea*	CT	https://doi.org/10.17602/M2/M117242
AMNH 203347	F	*Philander opossum*	Artec Spider	https://doi.org/10.17602/M2/M114646
AMNH 24232	F	*Philander opossum*	Artec Spider	https://doi.org/10.17602/M2/M114647
AMNH 61608	M	*Philander opossum*	Artec Spider	https://doi.org/10.17602/M2/M114648
AMNH 96714	M	*Philander opossum*	Artec Spider	https://doi.org/10.17602/M2/M114649
SMNS Z-MAM 21764	U	*Procyon lotor*	Artec Spider	https://doi.org/10.17602/M2/M115809
SMNS Z-MAM 51565	M	*Procyon lotor*	Artec Spider	https://doi.org/10.17602/M2/M115810
SMNS Z-MAM 51573	M	*Procyon lotor*	Artec Spider	https://doi.org/10.17602/M2/M115811
DMNH 2151	F	*Proteles cristata*	CT	https://doi.org/10.17602/M2/M114826
DMNH 2152	M	*Proteles cristata*	CT	https://doi.org/10.17602/M2/M114827
DMNH 37169	F	*Proteles cristata*	CT	https://doi.org/10.17602/M2/M114828

ZMB Mam 45001	M	*Proteles cristata*	Artec Spider	https://doi.org/10.17602/M2/M116019
ZMB Mam 13002	F	*Puma concolor*	Artec Spider	https://doi.org/10.17602/M2/M116020
ZMB Mam 14482	F	*Puma concolor*	Artec Spider	https://doi.org/10.17602/M2/M116021
ZMB Mam 37691	M	*Puma concolor*	Artec Spider	https://doi.org/10.17602/M2/M116022
ZMB Mam 72929	U	*Puma concolor*	Artec Spider	https://doi.org/10.17602/M2/M116023
NMV C 17712	M	*Sarcophilus harrisii*	Artec Spider	https://doi.org/10.17602/M2/M115145
NMV C 6251	M	*Sarcophilus harrisii*	Artec Spider	https://doi.org/10.17602/M2/M115150
WAM M 16592	F	*Sarcophilus harrisii*	Artec Spider	https://doi.org/10.17602/M2/M115889
WAM M 8336	F	*Sarcophilus harrisii*	Artec Spider	https://doi.org/10.17602/M2/M115890
SMNS Z-MAM 19136	U	*Speothos venaticus*	Artec Spider	https://doi.org/10.17602/M2/M115812
ZMB Mam 31003	U	*Speothos venaticus*	Artec Spider	https://doi.org/10.17602/M2/M116024
ZMB Mam 4680	F	*Speothos venaticus*	Artec Spider	https://doi.org/10.17602/M2/M116025
AM M 19465	M	*Thylacinus cynocephalus*	Artec Spider	https://doi.org/10.17602/M2/M114636
NHMUK ZD 1839.6.11.3	F	*Thylacinus cynocephalus*	Artec Spider	https://doi.org/10.17602/M2/M114679
NHMUK ZD 1846.4.4.1	M	*Thylacinus cynocephalus*	Artec Spider	https://doi.org/10.17602/M2/M114680
NHMUK ZD 1852.1.16.7	F	*Thylacinus cynocephalus*	Artec Spider	https://doi.org/10.17602/M2/M114681
NHMUK ZD 1972.665	M	*Thylacinus cynocephalus*	Artec Spider	https://doi.org/10.17602/M2/M114682
NHMUK ZD 1972.666	F	*Thylacinus cynocephalus*	Artec Spider	https://doi.org/10.17602/M2/M114683
NMV C 5742	F	*Thylacinus cynocephalus*	Artec Spider	https://doi.org/10.17602/M2/M115159
NMV C 5749	M	*Thylacinus cynocephalus*	Artec Spider	https://doi.org/10.17602/M2/M115160
SAM M 1953	M	*Thylacinus cynocephalus*	Artec Spider	https://doi.org/10.17602/M2/M115277
SAM M 1959	F	*Thylacinus cynocephalus*	Artec Spider	https://doi.org/10.17602/M2/M115278
SAM M 922	F	*Thylacinus cynocephalus*	Artec Spider	https://doi.org/10.17602/M2/M115279
SAM M 95	M	*Thylacinus cynocephalus*	Artec Spider	https://doi.org/10.17602/M2/M115280
TMAG 321	M	*Thylacinus cynocephalus*	Artec Spider	https://doi.org/10.17602/M2/M115876
UMZC A673	M	*Thylacinus cynocephalus*	Artec Spider	https://doi.org/10.17602/M2/M115877
UMZC A678	F	*Thylacinus cynocephalus*	Artec Spider	https://doi.org/10.17602/M2/M115878
WAM M 195	U	*Thylacinus cynocephalus*	Artec Spider	https://doi.org/10.17602/M2/M115891
AMNH 1298	M	*Urocyon cinereoargenteus*	Artec Spider	https://doi.org/10.17602/M2/M114650
AMNH 255648	F	*Urocyon cinereoargenteus*	Artec Spider	https://doi.org/10.17602/M2/M114651

AMNH 32302	F	*Urocyon cinereoargenteus*	Artec Spider	https://doi.org/10.17602/M2/M114652
NMV C 11222	M	*Urocyon cinereoargenteus*	Artec Spider	https://doi.org/10.17602/M2/M115161
SMNS Z-MAM 2257	F	*Viverra tangalunga*	Artec Spider	https://doi.org/10.17602/M2/M115814
SMNS Z-MAM 2507	U	*Viverra zibetha*	Artec Spider	https://doi.org/10.17602/M2/M115815
SMNS Z-MAM 21594	U	*Viverricula indica*	Artec Spider	https://doi.org/10.17602/M2/M115813
WAM 30616	U	*Viverricula indica*	Artec Spider	https://doi.org/10.17602/M2/M115892
ZMB Mam 103828	M	*Vulpes lagopus*	Artec Spider	https://doi.org/10.17602/M2/M116032
ZMB Mam 2205	F	*Vulpes lagopus*	Artec Spider	https://doi.org/10.17602/M2/M116033
ZMB Mam 65305	F	*Vulpes lagopus*	Artec Spider	https://doi.org/10.17602/M2/M116034
ZMB Mam 65324	M	*Vulpes lagopus*	Artec Spider	https://doi.org/10.17602/M2/M116035
DMNH 1193	M	*Vulpes chama*	CT	https://doi.org/10.17602/M2/M114829
DMNH 1493	M	*Vulpes chama*	CT	https://doi.org/10.17602/M2/M114830
ZMB Mam 25253	U	*Vulpes rueppellii*	Artec Spider	https://doi.org/10.17602/M2/M116026
ZMB Mam 49961	M	*Vulpes rueppellii*	Artec Spider	https://doi.org/10.17602/M2/M116027
ZMB Mam 6080	U	*Vulpes rueppellii*	Artec Spider	https://doi.org/10.17602/M2/M116028
ZMB Mam 91331	U	*Vulpes rueppellii*	Artec Spider	https://doi.org/10.17602/M2/M116029
NMV C 25076	M	*Vulpes vulpes*	Artec Spider	https://doi.org/10.17602/M2/M115162
SMNS Z-MAM 31949	M	*Vulpes vulpes*	Artec Spider	https://doi.org/10.17602/M2/M115816
ZMB Mam 43558	F	*Vulpes vulpes*	Artec Spider	https://doi.org/10.17602/M2/M116030
ZMB Mam 65666	F	*Vulpes vulpes*	Artec Spider	https://doi.org/10.17602/M2/M116031

Table S7. References for phylogenetic hypotheses presented in **Figure 8.**

Beck, R. M. D. & Taglioretti, M. L. A nearly complete juvenile skull of the marsupial *Sparassocynus derivatus* from the Pliocene of Argentina, the affinities of "Sparassocynids", and the diversification of opossums (Marsupialia; Didelphimorphia; Didelphidae). *Journal of Mammalian Evolution*, doi:10.1007/s10914-019-09471-y (2019).

Eizirik, E. *et al.* Pattern and timing of diversification of the mammalian order Carnivora inferred from multiple nuclear gene sequences. *Molecular Phylogenetics and Evolution* 56, 49-63, doi:10.1016/j.ympev.2010.01.033 (2010).

Johnson, W. E. *et al.* The late Miocene radiation of modern Felidae: A genetic assessment. *Science* 311, 73-77, doi:10.1126/science.1122277 (2006).

Kealy, S. & Beck, R. Total evidence phylogeny and evolutionary timescale for Australian faunivorous marsupials (Dasyuromorphia). *BMC Evolutionary Biology* 17, 1-23 (2017).

Koepfli, K.-P. *et al.* Molecular systematics of the Hyaenidae: relationships of a relictual lineage resolved by a molecular supermatrix. *Molecular Phylogenetics and Evolution* 38, 603-620 (2006).

Koepfli, K. *et al.* Genome-wide evidence reveals that African and Eurasian golden jackals are distinct species. *Current Biology* 25, 2158-2165 (2015).

Law, C. J., Slater, G. J. & Mehta, R. S. Lineage diversity and size disparity in Musteloidea: Testing patterns of adaptive radiation using molecular and fossil-based methods. *Systematic Biology* 67, 127-144, doi:10.1093/sysbio/syx047 (2018).

Mitchell, K. J. *et al.* Molecular phylogeny, biogeography, and habitat preference evolution of marsupials. *Molecular Biology and Evolution* 31, 2322-2330, doi:10.1093/molbev/msu176 (2014).

Perini, F. A., Russo, C. A. M. & Schrago, C. G. The evolution of South American endemic canids: a history of rapid diversification and morphological parallelism. *Journal of Evolutionary Biology* 23, 311-322, doi:10.1111/j.1420-9101.2009.01901.x (2010).

Piras, P. *et al.* Evolution of the sabertooth mandible: A deadly ecomorphological specialization. *Palaeogeography, Palaeoclimatology, Palaeoecology* 496, 166-174, doi:https://doi.org/10.1016/j.palaeo.2018.01.034 (2018).

Rovinsky, D. S., Evans, A. R. & Adams, J. W. The pre-Pleistocene fossil thylacinids (Dasyuromorphia: Thylacinidae) and the evolutionary context of the modern thylacine. *PeerJ* 7, e7457, doi:10.7717/peerj.7457 (2019).

Slater, G. J. & Friscia, A. R. Hierarchy in adaptive radiation: A case study using the Carnivora (Mammalia). *Evolution* 73, 524-539, doi:10.1111/evo.13689 (2019).

Slater, G. J., Harmon, L. J. & Alfaro, M. E. Integrating fossils with molecular phylogenies improves inference of trait evolution. *Evolution* 66, 3931-3944, doi:10.1111/j.1558-5646.2012.01723.x (2012).

Tchaicka, L. *et al.* Molecular assessment of the phylogeny and biogeography of a recently diversified endemic group of South American canids (Mammalia: Carnivora: Canidae). *Genetics and Molecular Biology* 39, 442-451, doi:10.1590/1678-4685-GMB-2015-0189 (2016).

Zhao, C., Zhang, H., Liu, G., Yang, X. & Zhang, J. The complete mitochondrial genome of the Tibetan fox (*Vulpes ferrilata*) and implications for the phylogeny of Canidae. *Comptes Rendus Biologies* 339, 68-77, doi:https://doi.org/10.1016/j.crvi.2015.11.005 (2016).

Zhou, Y., Wang, S.-R. & Ma, J.-Z. Comprehensive species set revealing the phylogeny and biogeography of Feliformia (Mammalia, Carnivora) based on mitochondrial DNA. *PLoS ONE* 12, e0174902, doi:10.1371/journal.pone.0174902 (2017).

Table S8. 3D GM landmark protocol and definitions.

Point	Definition	Type	Paired	Semi-landmarks
1	Anteriormost extent of premaxilla at midline (Prosthion)	Fixed	-	-
2	Anteriormost nasal (L)	Fixed	yes	-
3	Anteriormost nasal (R)	Fixed	yes	-
4	Anterior nasopremaxilla (L)	Fixed	yes	-
5	Anterior nasopremaxilla (R)	Fixed	yes	-
6	Mesial canine alveolar margin in lateral view (L)	Fixed	yes	-
7	Mesial canine alveolar margin in lateral view (R)	Fixed	yes	-
8	Distal canine alveolar margin in lateral view (L)	Fixed	yes	-
9	Distal canine alveolar margin in lateral view (R)	Fixed	yes	-
10	Midline at point coplanar with & dorsal to 8/9	Fixed	-	-
11	Midline at posterior edge of incisive foramen	Fixed	-	-
12	Distal alveolar margin of P3 in lateral view (L)	Fixed	yes	-
13	Distal alveolar margin of P3 in lateral view (R)	Fixed	yes	-
14	Dorsal border of infraorbital foramen (L)	Fixed	yes	-
15	Dorsal border of infraorbital foramen (R)	Fixed	yes	-
16	Ventral border of infraorbital foramen (L)	Fixed	yes	-
17	Ventral border of infraorbital foramen (R)	Fixed	yes	-
18	Mesial alveolar margin of carnassial in lateral view (L)	Fixed	yes	-
19	Mesial alveolar margin of carnassial in lateral view (R)	Fixed	yes	-
20	Distal alveolar margin of carnassial in lateral view (L)	Fixed	yes	-
21	Distal alveolar margin of carnassial in lateral view (R)	Fixed	yes	-
22	Postorbital process (L)	Fixed	yes	-
23	Postorbital process (R)	Fixed	yes	-
24	Midline at point in line with 22/23	Fixed	-	-
25	Postorbital process of zygomatic (R)	Fixed	yes	-
26	Postorbital process of zygomatic (L)	Fixed	yes	-
27	Ventrodistal inflection of jugal with maxilla (R)	Fixed	yes	-
28	Ventrodistal inflection of jugal with maxilla (L)	Fixed	yes	-
29	Midline at distalmost interpalatine	Fixed	-	-
30	Anterolateral point of glenoid on squamosal (L)	Fixed	yes	-
31	Anterolateral point of glenoid on squamosal (R)	Fixed	yes	-
32	Anteromedial point of glenoid on squamosal (L)	Fixed	yes	-
33	Anteromedial point of glenoid on squamosal (R)	Fixed	yes	-
34	Midline of basicranium at anterior extent of auditory bullae	Fixed	-	-
35	Posterior inflection of zygomatic with neurocranium (R)	Fixed	yes	-
36	Posterior inflection of zygomatic with neurocranium (L)	Fixed	yes	-
37	Lateralmost point of nuchal crest (R)	Fixed	yes	-
38	Lateralmost point of nuchal crest (L)	Fixed	yes	-
39	Ventralmost point of mastoid process (R)	Fixed	yes	-
40	Ventralmost point of mastoid process (L)	Fixed	yes	-
41	Ventralmost point of paracondylar process (R)	Fixed	yes	-
42	Ventralmost point of paracondylar process (L)	Fixed	yes	-
43	Ventral border of foramen magnum at midline (Basion)	Fixed	-	-
44	Dorsal border of foramen magnum at midline (Opisthion)	Fixed	-	-
45	Confluence of temporal and nuchal crests (R)	Fixed	yes	-
46	Confluence of temporal and nuchal crests (L)	Fixed	yes	-
C1	Curve between points 10 & 26 (dorsum of rostrum)	Curve	-	9

C2	Curve between points 11 % 29 (palatal curve)	Curve	-	9
C3	Curve between points 9 & 10 (rostral arc at canine)	Curve	-	5
C4	Curve between points 8 & 10 (rostral arc at canine)	Curve	-	5
C5	Curve between points 9 & 13 (alveolar margin)	Curve	-	5
C6	Curve between points 8 & 12 (alveolar margin)	Curve	-	5
C7	Curve between points 23 & 25 (orbital rim R)	Curve	-	11
C8	Curve between points 22 & 26 (orbital rim L)	Curve	-	11
C9	Curve between point 15 & anterior rim of orbit (dorsal infraorbital foramen R)	Curve	-	3
C10	Curve between point 14 & anterior rim of orbit (dorsal infraorbital foramen R)	Curve	-	3
C11	Curve between points 17 & 13 (ventral infraorbital foramen R)	Curve	-	3
C12	Curve between points 16 & 12 (ventral infraorbital foramen L)	Curve	-	3
C13	Curve between points 25 & 35 (dorsal zygomatic border R)	Curve	-	13
C14	Curve between points 26 & 36 (dorsal zygomatic border L)	Curve	-	13
C15	Curve between points 27 & 31 (ventral zygomatic border R)	Curve	-	11
C16	Curve between points 28 & 30 (ventral zygomatic border L)	Curve	-	11
C17	Curve between points 31 & 33 (anterior border of glenoid R)	Curve	-	5
C18	Curve between points 30 & 32 (anterior border of glenoid L)	Curve	-	5
C19	Curve between points 34 & 43 (basicranial curve)	Curve	-	3
C20	Curve between 22 & 46 (temporal crest L)	Curve	-	13
C21	Curve between 23 & 45 (temporal crest R)	Curve	-	13
C22	Curve between 38 & 46 (nuchal crest L)	Curve	-	9
C23	Curve between 37 & 45 (nuchal crest R)	Curve	-	9
C24	Curve between 22 & 32 (infratemporal crest L)	Curve	-	7
C25	Curve between 23 & 33 (infratemporal crest R)	Curve	-	7
P1	Patch bordered by c1, c3, c5, c7, c9, c11 (rostal patch R)	Patch	-	36
P2	Patch bordered by c1, c4, c6, c8, c10, c12 (rostral patch L)	Patch	-	36
P3	Patch bordered by c21, c23, c25 (neurocranial patch R)	Patch	-	36
P4	Patch bordered by c20, c22, c24 (neurocranial patch L)	Patch	-	36

Detailed protocol:

Surface meshes were imported into Geomagic Studio 2014 and oriented via the sagittal plane and a horizontal plane defined by the dorsalmost point of the right auditory meatus and the ventralmost point of the left and right orbital rim, producing an orientation similar to Frankfort horizontal (FH). This orientation allowed for placement of functionally analogous fixed landmarks and curve semilandmarks at geometrically definable positions relative to FH. Landmarking of all specimens was performed by DSR in Viewbox 4 (dHAL software, Greece). A landmark template was produced and then used to semi-automate the placement and projection of curve and patch semilandmarks via thin-plate spline warping of the template to the target mesh. A mean landmark configuration was generated from the total dataset, and the semilandmarks of each specimen was then slid on a tangent to the surface or curve spline to minimise bending energy in reference to the mean configuration. The semilandmarks were then projected back onto their respective mesh surface, and the sliding process was repeated six times, further minimising bending energy with each repetition. This procedure was performed five more times, each time generating a mean landmark configuration from the previous iteration, then sliding and projecting onto the respective surface mesh six times. The resulting configurations were then considered to be homologous, and used in the subsequent Procrustes superimposition.

Table S9. References for diet, body mass, and prey size. Data can be found on Figshare at https://figshare.com/projects/Convergence_between_the_thylacine_and_small_prey-focused_canids/84905

Abreu KC, Moro-Rios RF, Silva-Pereira JE, Miranda JM, Jablonski EF, and Passos FC. 2008. Feeding habits of ocelot (*Leopardus pardalis*) in Southern Brazil. *Mammalian Biology* 73:407-411. 10.1016/j.mambio.2007.07.004

Andelt WF, Kie JG, Knowlton FF, and Cardwell K. 1987. Variation in coyote diets associated with season and successional changes in vegetation. *Journal of Wildlife Management* 51:273-277.

Angelici FM. 2000. Food habits and resource partitioning of carnivores (Herpestidae, Viverridae) in the rainforests of southeastern Nigeria: preliminary results. *Revue d'Écologie* 55:67-76.

Angelici FM, and Luiselli L. 2005. Habitat associations and dietary relationships between two genets, *Genetta maculata* and *Genetta cristata*. *Revue d'Écologie* 60:341-354.

Anthony RM, Barten NL, and Seiser PE. 2000. Foods of arctic foxes (*Alopex lagopus*) during winter and spring in western Alaska. *Journal of Mammalogy* 81:820-828.

Anwar MB, Jackson R, Nadeem MS, Janečka JE, Hussain S, Beg MA, Muhammad G, and Qayyum M. 2011. Food habits of the snow leopard *Panthera uncia* (Schreber, 1775) in Baltistan, Northern Pakistan. *European Journal of Wildlife Research* 57:1077-1083. 10.1007/s10344-011-0521-2

Atkinson RPD, Macdonald DW, and Kamizola R. 2002. Dietary opportunism in side-striped jackals *Canis adustus* Sundevall. *Journal of Zoology* 257:129-139.

Azevedo F, Lester V, Gorsuch W, Lariviere S, Wirsing A, and Murray D. 2006. Dietary breadth and overlap among five sympatric prairie carnivores. *Journal of Zoology* 269:127-135. 10.1111/j.1469-7998.2006.00075.x

Baker CM. 1989. Feeding habits of the water mongoose (*Atilax paludinosus*). *Zeitschrift für Säugetierkunde* 54:31-39.

Belcher CA. 1995. Diet of the tiger quoll (*Dasyurus maculatus*) in East Gippsland, Victoria. *Wildlife Research* 22:341-357.

Blackhall S. 1980. Diet of the eastern native-cat, *Dasyurus viverrinus* (Shaw), in southern Tasmania. *Wildlife Research* 7:191-197.

Bothma JdP. 1966. Food of the silver fox *Vulpes chama*. *Zoologica Africana* 2:205-209.

Bowland JM, and Perrin M. 1993. Diet of serval *Felis serval* in a highland region of Natal. *African Zoology* 28:132-135.

Braczkowski A, Watson L, Coulson D, Lucas J, Peiser B, and Rossi M. 2012. The diet of caracal, *Caracal caracal*, in two areas of the southern Cape, South Africa as determined by scat analysis. *South African Journal of Wildlife Research* 42:111-116.

Cáceres N, Ghizoni-Jr I, and Graipel M. 2002. Diet of two marsupials, *Lutreolina crassicaudata* and *Micoureus demerarae*, in a coastal Atlantic Forest island of Brazil. *Mammalia* 66:331-340.

Capitani C, Bertelli I, Varuzza P, Scandura M, and Apollonio M. 2004. A comparative analysis of wolf (*Canis lupus*) diet in three different Italian ecosystems. *Mammalian Biology* 69:1-10.

Castelló JR. 2018. *Canids of the world: wolves, wild dogs, foxes, jackals, coyotes, and their relatives*. Princeton: Princeton University Press.

Castillo DF, Birochio DE, Lucherini M, and Casanave EB. 2011. Diet of adults and cubs of *Lycalopex gymnocercus* in Pampas grassland: a validation of the Optimal Foraging Theory? *Annales Zoologici Fennici* 48:251-256.

Castro-Arellano I, Zarza H, and Medellín RA. 2000. *Philander opossum*. *Mammalian Species* 2000:1-8.

Chetri M, Odden M, and Wegge P. 2017. Snow leopard and Himalayan wolf: food habits and prey selection in the Central Himalayas, Nepal. *PLoS ONE* 12:1-16. 10.1371/journal.pone.0170549

Chuang SA, and Lee LL. 1997. Food habits of three carnivore species (*Viverricula indica*, *Herpestes urva*, and *Melogale moschata*) in Fushan Forest, northern Taiwan. *Journal of Zoology* 243:71-79.

Clevenger A. 1993. Pine marten (*Martes martes* Linné, 1758) comparative feeding ecology in an island and mainland population of Spain. *Zeitschrift für Säugetierkunde* 58:212-212.

Colon CP, and Sugau JB. 2012. Notes on the diet of the Malay civet (*Viverra tangalunga*) and other civets in logged and unlogged lowland dipterocarp rain forests in Sabah, Borneo. *Malayan Nature Journal* 64:69-74.

Cooper R, and Skinner J. 1979. Importance of termites in the diet of the aardwolf *Proteles cristatus* in South Africa. *South African Journal of Zoology* 14:5-8.

Cordero G, and Nicolas R. 1987. Feeding habits of the opossum (*Didelphis marsupialis*) in northern Venezuela. *Fieldiana Zoology*:125-131.

Courchamp F, Rasmussen GSA, and Macdonald DW. 2002. Small pack size imposes a trade-off between hunting and pup-guarding in the painted hunting dog *Lycaon pictus*. *Behavioral Ecology* 13:20-27. 10.1093/beheco/13.1.20

Creel S, and Creel NM. 1995. Communal hunting and pack size in African wild dogs, *Lycaon pictus*. *Animal Behaviour* 50:1325-1339.

Dalerum F, Kunkel K, Angerbjörn A, and Shults BS. 2009. Diet of wolverines (*Gulo gulo*) in the western Brooks Range, Alaska. *Polar Research* 28:246-253. 10.1111/j.1751-8369.2008.00090.x

de Oliveira T. 2002. Ecología comparativa de la alimentación del jaguar y del puma en el neotrópico. In: Medellín RA, Equihua C, Chetkiewicz C, Crawshaw Jr P, Rabinowitz A, Redford K, Robinson J, Sanderson E, and Taber A, eds. *El jaguar en el nuevo milenio*. Mexico City: Fondo de Cultura Económica, 265-288.

de Souza Lima E, Jorge RSP, and Dalponte JC. 2009. Habitat use and diet of bush dogs, *Speothos venaticus*, in the Northern Pantanal, Mato Grosso, Brazil. *Mammalia* 73:13-19. 10.1515/MAMM.2009.002

de Villa Meza A, Martinez Meyer E, and López González C. 2002. Ocelot (*Leopardus pardalis*) food habits in a tropical deciduous forest of Jalisco, Mexico. *American Midland Naturalist* 148:146-154.

Dollar L, Ganzhorn JU, and Goodman SM. 2007. Primates and other prey in the seasonally variable diet of *Cryptoprocta ferox* in the dry deciduous forest of western Madagascar. In: Gursky SL, and Nekaris KAI, eds. *Primate anti-predator strategies*. Boston: Springer, 63-76.

Facure KG, and do Nascimento Ramos V. 2011. Food habits of the thick-tailed opossum Lutreolina crassicaudata (Didelphimorphia, Didelphidae) in two urban areas of southeastern Brazil. *Mammalian Biology* 76:234-236. 10.1016/j.mambio.2010.06.005

Farrell LE, Roman J, and Sunquist ME. 2000. Dietary separation of sympatric carnivores identified by molecular analysis of scats. *Molecular Ecology* 9:1583-1590.

Gade-Jørgensen I, and Stagegaard R. 2000. Diet composition of wolves *Canis lupus* in east-central Finland. *Acta Theriologica* 45:537-547.

Galliez M, de Souza Leite M, Queiroz TL, and dos Santos Fernandez FA. 2009. Ecology of the water opossum *Chironectes minimus* in Atlantic forest streams of southeastern Brazil. *Journal of Mammalogy* 90:93-103.

Giannatos G, Karypidou A, Legakis A, and Polymeni R. 2010. Golden jackal (*Canis aureus* L.) diet in Southern Greece. *Mammalian Biology* 75:227-232. 10.1016/j.mambio.2009.03.003

Gilbert F, and Nancekivell E. 1982. Food habits of mink (*Mustela vison*) and otter (*Lutra canadensis*) in northeastern Alberta. *Canadian Journal of Zoology* 60:1282-1288.

Glen A, and Dickman C. 2006. Diet of the spotted-tailed quoll (*Dasyurus maculatus*) in eastern Australia: effects of season, sex and size. *Journal of Zoology* 269:241-248. 10.1111/j.1469-7998.2006.00046.x

Godsell J. 1983. Ecology of the eastern quoll *Dasyurus viverrinus*, (Dasyrudae: Marsupialia) Doctor of Philosophy. Australian National University.

Graw B, and Manser M. 2017. Life history patterns and biology of the slender mongoose (*Galerella sanguinea*) in the Kalahari Desert. *Journal of Mammalogy* 98:332-338. 10.1093/jmammal/gyw178

Hayward M. 2006. Prey preferences of the spotted hyaena (*Crocuta crocuta*) and degree of dietary overlap with the lion (*Panthera leo*). *Journal of Zoology* 270:606-614. 10.1111/j.1469-7998.2006.00183.x

Hayward M, Hofmeyr M, O'brien J, and Kerley G. 2006. Prey preferences of the cheetah (*Acinonyx jubatus*)(Felidae: Carnivora): morphological limitations or the need to capture rapidly consumable prey before kleptoparasites arrive? *Journal of Zoology* 270:615-627. 10.1111/j.1469-7998.2006.00184.x

Hayward MW, Lyngdoh S, and Habib B. 2014. Diet and prey preferences of dholes (*Cuon alpinus*): dietary competition within Asia's apex predator guild. *Journal of Zoology* 294:255-266. 10.1111/jzo.12171

Henschel P, Abernethy K, and White L. 2005. Leopard food habits in the Lopé National Park, Gabon, Central Africa. *African Journal of Ecology* 43:21-28.

Hershkovitz P. 1997. Composition of the family Didelphidae Gray, 1821 (Didelphoidea: Marsupialia), with a review of the morphology and behavior of the included four-eyed pouched opossums of the genus *Philander* Tiedemann, 1808. *Fieldiana Zoology* 86:1-103.

Hockman JG, and Chapman JA. 1983. Comparative feeding habits of red foxes (*Vulpes vulpes*) and gray foxes (*Urocyon cinereoargenteus*) in Maryland. *American Midland Naturalist* 110:276-285. 10.2307/2425269

Holekamp KE, Smale L, Berg R, and Cooper SM. 1997. Hunting rates and hunting success in the spotted hyena (*Crocuta crocuta*). *Journal of Zoology* 242:1-15. 10.1111/j.1469-7998.1997.tb02925.x

Iriarte JA, Franklin WL, Johnson WE, and Redford KH. 1990. Biogeographic variation of food habits and body size of the America puma. *Oecologia* 85:185-190. 10.1007/BF00319400

Jędrzejewski W, and Jędrzejewska B. 1992. Foraging and diet of the red fox *Vulpes vulpes* in relation to variable food resources in Białowieza National Park, Poland. *Ecography* 15:212-220. 10.1111/j.1600-0587.1992.tb00027.x

Johnsingh AJT. 1982. Reproductive and social behaviour of the Dhole, *Cuon alpinus* (Canidae). *Journal of Zoology* 198:443-463. 10.1111/jzo.1982.198.4.443

Johnson KG, Wei W, Reid DG, and Jinchu H. 1993. Food Habits of Asiatic Leopards (*Panthera pardus fusea*) in Wolong Reserve, Sichuan, China. *Journal of Mammalogy* 74:646-650. 10.2307/1382285

Jones ME. 1997. Character displacement in Australian dasyurid carnivores: size relationships and prey size patterns. *Ecology* 78:2569-2587.

Jones ME, and Barmuta LA. 1998. Diet overlap and relative abundance of sympatric dasyurid carnivores: a hypothesis of competition. *Journal of Animal Ecology* 67:410-421.

Kitchen AM, Gese EM, and Schauster ER. 1999. Resource partitioning between coyotes and swift foxes: space, time, and diet. *Canadian Journal of Zoology* 77:1645-1656. 10.1139/z99-143

Klare U, Kamler JF, and Macdonald DW. 2011. The bat-eared fox: A dietary specialist? *Mammalian Biology* 76:646-650. 10.1016/j.mambio.2011.06.005

Klare U, Kamler JF, and Macdonald DW. 2014. Seasonal diet and numbers of prey consumed by Cape foxes *Vulpes chama* in South Africa. *Wildlife Biology* 20:190-195. 10.2981/wlb.00006

Koskela A, Kojola I, Aspi J, and Hyvärinen M. 2013. The diet of breeding female wolverines (*Gulo gulo*) in two areas of Finland. *Acta Theriologica* 58:199-204. 10.1007/s13364-012-0111-z

Landa A, Strand O, Swenson JE, and Skogland T. 1997. Wolverines and their prey in southern Norway. *Canadian Journal of Zoology* 75:1292-1299. 10.1139/z97-153

Lanszki J, and Heltai M. 2002. Feeding habits of golden jackal and red fox in south-western Hungary during winter and spring. *Mammalian Biology* 67:129-136. 10.1078/1616-5047-00020

Larivière S, and Seddon PJ. 2001. *Vulpes rueppelli*. *Mammalian Species* 678:1-5. 10.1644/1545-1410(2001)678<0001:VR>2.0.CO;2

Lindsay IM, and Macdonald DW. 1986. Behaviour and ecology of the Ruppell's fox, *Vulpes ruppelli*, in Oman. *Mammalia* 50:461-474. https://doi.org/10.1515/mamm.1986.50.4.461

Loveridge AJ, and Macdonald DW. 2003. Niche separation in sympatric jackals (*Canis mesomelas* and *Canis adustus*). *Journal of Zoology* 259:143-153. 10.1017/S0952836902003114

Marshall LG. 1978. *Chironectes minimus*. *Mammalian Species*:1-6. 10.2307/0.109.1

McShane TO, and Grettenberger JF. 1984. Food of the golden jackal (*Canis aureus*) in central Niger. *African Journal of Ecology* 22:49-53. 10.1111/j.1365-2028.1984.tb00673.x

Medellín RA. 1991. Ecomorfología del cráneo de cinco didélfidos: tendencias, divergencias e implicaciones. *Anales del Instituto de Biología de la Universidad Nacional Autónoma de México, Serie Zoología* 62:269-286.

Melville HIAS, Bothma JdP, and Mills MGL. 2004. Prey selection by caracal in the Kgalagadi Transfrontier Park. *South African Journal of Wildlife Research* 34:67-75.

Moreno RS, Kays RW, and Samudio R, Jr. 2006. Competitive release in diets of ocelot (*Leopardus pardalis*) and puma (*Puma concolor*) after jaguar (*Panthera onca*) decline. *Journal of Mammalogy* 87:808-816. 10.1644/05-mamm-a-360r2.1

Motta-Junior JC, Talamoni SA, Lombardi JA, and Simokomaki K. 1996. Diet of the maned wolf, *Chrysocyon brachyurus*, in central Brazil. *Journal of Zoology* 240:277-284. 10.1111/j.1469-7998.1996.tb05284.x

Newsome A, Corbett L, Catling P, and Burt R. 1983. The feeding ecology of the dingo. 1. Stomach contents from trapping in south-eastern Australia, and the non-target wildlife also caught in dingo traps. *Wildlife Research* 10:477-486. 10.1071/WR9830477

Oakwood M. 1997. The ecology of the northern quoll, *Dasyurus hallucatus* Doctor of Philosophy. Australian National University.

Odden J, Linnell JDC, and Andersen R. 2006. Diet of Eurasian lynx, *Lynx lynx*, in the boreal forest of southeastern Norway: the relative importance of livestock and hares at low roe deer density. *European Journal of Wildlife Research* 52:237-244. 10.1007/s10344-006-0052-4

Owens MJ, and Owens DD. 1978. Feeding ecology and its influence on social organization in Brown hyenas (*Hyaena brunnea*, Thunberg) of the Central Kalahari Desert. *African Journal of Ecology* 16:113-135. 10.1111/j.1365-2028.1978.tb00433.x

Palmer R, and Fairall N. 1988. Caracal and African wild cat diet in the Karoo National Park and the implications thereof for hyrax. *South African Journal of Wildlife Research* 18:30-34.

Paltridge R. 2002. The diets of cats, foxes and dingoes in relation to prey availability in the Tanami Desert, Northern Territory. *Wildlife Research* 29:389-403. 10.1071/WR00010

Pemberton DA, Gales SB, B, Gales R, Lazenby B, and Medlock K. 2008. The diet of the Tasmanian Devil, *Sarcophilus harrisii*, as determined from analysis of scat and stomach contents. *Papers and Proceedings of the Royal Society of Tasmania* 142:13-22. 10.26749/rstpp.142.2.13

Pollock A. 1999. Notes on status, distribution and diet of northern quoll *Dasyurus hallucatus* in the Mackay-Bowen area, mideastern Queensland. *Australian Zoologist* 31:388-395.

Rabinowitz AR, and Nottingham Jr BG. 1986. Ecology and behaviour of the Jaguar (*Panthera onca*) in Belize, Central America. *Journal of Zoology* 210:149-159. 10.1111/j.1469-7998.1986.tb03627.x

Reed JE, Ballard WB, Gipson PS, Kelly BT, Krausman PR, Wallace MC, and B. WD. 2006. Diets of free-ranging Mexican gray wolves in Arizona and New Mexico. *Wildlife Society Bulletin* 34:1127-1133. 10.2193/0091-7648(2006)34[1127:dofmgw]2.0.co;2

Schaller GB, and Vasconcelos JMC. 1978. Jaguar predation on capybara. *Zeitschrift für Säugetierkunde* 43:296-301.

Schoonover LJ, and Marshall WH. 1951. Food habits of the raccoon (*Procyon lotor hirtus*) in north-central Minnesota. *Journal of Mammalogy* 32:422-428. 10.2307/1375790

Sidorovich VE, Sidorovich AA, and Izotova IV. 2006. Variations in the diet and population density of the red fox *Vulpes vulpes* in the mixed woodlands of northern Belarus. *Mammalian Biology* 71:74-89. 10.1016/j.mambio.2005.12.001

Sillero-Zubiri C, and Gottelli D. 1994. *Canis simensis. Mammalian Species*:1-6. 10.2307/3504136

Sillero-Zubiri C, Hoffmann M, and Macdonald DW. 2004. *Canids: foxes, wolves, jackals, and dogs: status survey and conservation action plan*: IUCN Gland, Switzerland.

Soderquist T, and Serena M. 1994. Dietary niche of the western quoll, *Dasyurus geoffroii*, in the jarrah forest of Western Australia. *Australian Mammalogy* 17:133-136.

Stenglein JL, Waits LP, Ausband DE, Zager P, and Mack CM. 2011. Estimating gray wolf pack size and family relationships using noninvasive genetic sampling at rendezvous sites. *Journal of Mammalogy* 92:784-795. 10.1644/10-mamm-a-200.1

Tófoli C, Rohe F, and Setz E. 2009. Jaguarundi (*Puma yagouaroundi*) (Geoffroy, 1803) (Carnivora, Felidae) food habits in a mosaic of Atlantic Rainforest and eucalypt plantations of southeastern Brazil. *Brazilian Journal of Biology* 69:871-877. 10.1590/S1519-69842009000400015

Valenzuela AEJ, Raya Rey A, Fasola L, Sáenz Samaniego RA, and Schiavini A. 2013. Trophic ecology of a top predator colonizing the southern extreme of South America: Feeding habits of invasive American mink (*Neovison vison*) in Tierra del Fuego. *Mammalian Biology* 78:104-110. 10.1016/j.mambio.2012.11.007

Varela O, Cormenzana-Méndez A, Krapovickas L, and Bucher EH. 2008. Seasonal diet of the Pampas fox (*Lycalopex gymnocercus*) in the Chaco dry woodland, northwestern Argentina. *Journal of Mammalogy* 89:1012-1019. 10.1644/07-mamm-a-125.1

Walton LR, and Joly DO. 2003. *Canis mesomelas*. *Mammalian Species* 2003:1-9.

Whitehouse SJO. 1977. The diet of the dingo in Western Australia. *Wildlife Research* 4:145-150. 10.1071/WR9770145

Zapata SC, Travaini A, Delibes M, and Martínez-Peck R. 2005. Food habits and resource partitioning between grey and culpeo foxes in southeastern Argentine Patagonia. *Studies on Neotropical Fauna and Environment* 40:97-103. 10.1080/01650520500129836

Zuercher GL, Gipson PS, and Carrillo O. 2005. Diet and habitat associations of bush dogs *Speothos venaticus* in the Interior Atlantic Forest of eastern Paraguay. *Oryx* 39:86-89. 10.1017/S0030605305000153
